# Topological DNA-binding of structural maintenance of chromosomes-like RecN promotes DNA double-strand break repair in *Escherichia coli*

**DOI:** 10.1038/s42003-019-0655-4

**Published:** 2019-11-14

**Authors:** Kenji Keyamura, Takashi Hishida

**Affiliations:** Department of Molecular Biology, Graduate School of Science, Gakushuin University, 1-5-1 Mejiro, Toshima-ku, Tokyo, 171-8588 Japan

**Keywords:** Double-strand DNA breaks, DNA recombination, DNA

## Abstract

Bacterial RecN, closely related to the structural maintenance of chromosomes (SMC) family of proteins, functions in the repair of DNA double-strand breaks (DSBs) by homologous recombination. Here we show that the purified *Escherichia coli* RecN protein topologically loads onto both single-stranded DNA (ssDNA) and double-stranded DNA (dsDNA) that has a preference for ssDNA. RecN topologically bound to dsDNA slides off the end of linear dsDNA, but this is prevented by RecA nucleoprotein filaments on ssDNA, thereby allowing RecN to translocate to DSBs. Furthermore, we found that, once RecN is recruited onto ssDNA, it can topologically capture a second dsDNA substrate in an ATP-dependent manner, suggesting a role in synapsis. Indeed, RecN stimulates RecA-mediated D-loop formation and subsequent strand exchange activities. Our findings provide mechanistic insights into the recruitment of RecN to DSBs and sister chromatid interactions by RecN, both of which function in RecA-mediated DSB repair.

## Abstract (web summary)

Kenji Keyamura and Takashi Hishida demonstrate that RecN binds to single-stranded and double-stranded DNA through topological entrapment and stimulates RecA-mediated strand exchange activity in bacteria. The results provide mechanistic insights into how RecN is recruited to and repairs DNA double-strand breaks.

## Introduction

DNA double-strand breaks (DSBs) are a major threat to genome stability and cell survival, and if left unrepaired or repaired inappropriately, can lead to genome rearrangements, aneuploidy, or cell death. Homologous recombination (HR) is highly conserved in all organisms and plays a critical role in DSB repair^1,2^. In *Escherichia coli*, DSBs are initially processed by the RecBCD helicase/nuclease to generate 3′ single-stranded DNA (ssDNA) tails, onto which RecA can assemble as a nucleoprotein filament^3–5^. The RecA filament then mediates homologous pairing, strand invasion, and strand exchange to form a joint molecule comprising the broken DNA and an intact homologous template^6^. In addition, the RecA nucleoprotein filament stimulates autocleavage of the LexA repressor and subsequent derepression of the SOS regulon, resulting in the induction of gene products involved in multiple cellular functions, such as DNA repair and cell division^7–10^. Thus, RecA is the central player in HR-dependent DSB repair and the RecA-mediated intermolecular interaction between sister chromatids must be carefully controlled to promote DSB repair and to avoid inappropriate recombination.

*E. coli recN*, a typical SOS-regulated gene, encodes a protein with a central coiled-coil domain and N- and C-terminal globular domains that contain Walker A and B motifs, respectively, which are characteristic of the structural maintenance of chromosomes (SMC) protein family^11–16^. These members play diverse roles in controlling chromosomal dynamics and integrity, including sister chromatid cohesion, chromosome condensation, and DNA repair^17–23^. *E. coli recN* mutants are highly sensitive to ionizing radiation, I-*Sce*I cleavage and mitomycin C (MMC), but not UV irradiation, and exhibit an abnormal morphology characterized by nucleoid fragmentation in the presence of MMC^24–29^, suggesting that RecN plays a specific role in DSB repair. We demonstrated previously that GFP-labeled RecN localizes in nucleoid-associated foci following DNA damage, and this recruitment is dependent on RecA^24^. A recent analysis by Vickridge et al. revealed that RecN maintains sister chromatid interactions during MMC treatment^30^. Taken together, these results suggest that RecA facilitates the loading of RecN onto DSB sites, and that RecN plays structural and functional roles in RecA-mediated DSB repair, possibly by maintaining the proximity of sister chromatids.

*E. coli* RecN has been difficult to purify because of its poor solubility^24,31^; hence the precise biochemical function of the protein remains unclear. In vitro studies of several other bacterial RecN proteins have revealed DNA-binding and DNA-stimulated ATPase activities^15,32–34^. In addition, like cohesin, RecN can promote the intermolecular ligation of linear double-stranded DNA (dsDNA) fragments^15,32,35^. A recent study showed that *Deinococcus radiodurans* RecN stimulates RecA-mediated HR in vitro^36^. These results imply that a SMC-like function of RecN might be involved in RecA-mediated DSB repair. However, the mechanism by which RecN is recruited to the sites of DSBs in a RecA-dependent manner, and how it associates simultaneously with two distinct DNA molecules during DSB repair, are still poorly understood.

In this study, we performed a biochemical characterization of *E. coli* RecN and found that it binds to ssDNA and dsDNA through topological entrapment. RecN topologically bound to dsDNA translocates from its initial binding site by sliding along the dsDNA until it reaches the ssDNA region, where the RecA nucleoprotein filament is already established. The RecA filament prevents the dissociation of RecN from the ends of DSBs via a physical interaction and/or serving as a structural impediment, allowing RecN to accumulate at the sites of DSBs. Furthermore, we found that RecN loaded onto ssDNA, but not dsDNA, captures a second dsDNA molecule, which is stimulated by ATP. RecN consistently stimulated RecA-mediated D-loop formation and subsequent strand exchange activity. Overall, our results demonstrate that, by topologically linking sister chromatids in close proximity, RecN promotes RecA-mediated synapsis during DSB repair.

## Results

### DNA-binding activity of RecN

In our initial experiment, we found that the ionic strength of the buffer impacts the solubility of *E. coli* RecN; subsequently, we were able to purify it to homogeneity as a histidine-tagged protein (His-RecN) using buffers containing 1 M NaCl (Supplementary Fig. 1a). The purified protein was relatively stable for months under low salt conditions. Gel filtration chromatography revealed that His-RecN was eluted as a single peak at a position indicating a molecular mass of 308 kDa, indicating that the purified protein exists in a homo‐oligomeric complex, most likely a tetramer (Supplementary Fig. 1a). Control experiments established that SOS-regulated *His-recN* when expressed from the multicopy plasmids fully complemented the repair deficiency of a ∆*recN* strain following exposure to MMC, indicating that the tagged protein functions similarly to wild-type RecN in vivo (Supplementary Fig. 1b).

**Fig. 1:**
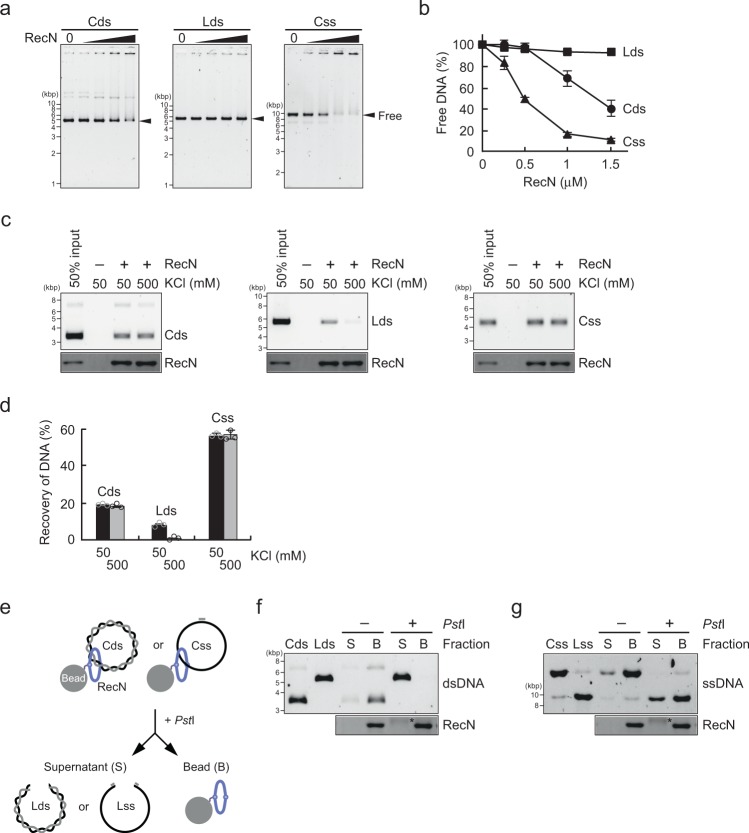
RecN topologically binds to DNA. **a** Electrophoretic mobility shift assays in which RecN (0–1.5 µM) was incubated at 37 °C for 10 min in the presence of the following phiX174-derivative DNA substrates: circular dsDNA (Cds), linear dsDNA (Lds), and circular ssDNA (Css). The samples were analyzed by agarose gel electrophoresis and SYBR Gold staining. **b** Quantification of the protein-free DNA bands in the gel images shown in **a**. The amount of free DNA (%) in each sample was normalized to that detected in the absence of RecN. Data represent the mean ± standard deviation of three independent experiments. **c** RecN (0.5 µM) was incubated at 37 °C for 10 min in the presence of the indicated phiX174-derivative DNA substrates. The RecN–DNA complexes were collected using Co^2+^-conjugated beads, washed in buffer containing 50 or 500 mM KCl, and eluted with SDS–sample buffer. The eluted proteins were analyzed by SDS–PAGE and Coomassie Brilliant Blue (CBB) staining, and the eluted DNA was analyzed by agarose gel electrophoresis and SYBR Gold staining. **d** Quantification of the intensities of the DNA bands in the agarose gel images shown in **c**. Data represent the mean ± standard deviation of three independent experiments. **e** Schematic illustration of DNA release by DNA linearization in **f**, **g**. **f** The RecN–Cds complex was isolated using the same method described above, and then incubated with or without *Pst*I. The bead (B) and supernatant (S) fractions were prepared and analyzed by agarose gel electrophoresis and SDS–PAGE as described above. Cds and Lds are shown as molecular size markers. **g** The assay was performed as described for **f** with the exception that Css annealed to a short oligonucleotide including a *Pst*I site was used as the substrate. Css and Lss (linear ssDNA) are shown as molecular markers. **f**, **g** The asterisks indicate bovine serum albumin derived from the *Pst*I stock solution

To determine whether *E. coli* RecN has a putative DNA-binding activity, circular dsDNA, linear dsDNA, and circular ssDNA substrates were incubated with increasing concentrations of RecN in the presence of ATP and the resulting complexes were separated by agarose gel electrophoresis. Electrophoretic mobility shift assays (EMSAs) revealed that RecN did not bind to linear dsDNA, but bound to circular dsDNA and ssDNA to form large protein–DNA complexes that did not enter the gel and preferentially bound to circular ssDNA rather than circular dsDNA (Fig. 1a, b).

Next, we examined formation of the RecN–DNA complex further using a pull-down assay with His-RecN. Following incubation of the DNA substrates described above with His-RecN, the protein–DNA complexes were collected using Co^2+^-conjugated magnetic beads and analyzed by agarose gel electrophoresis (for RecN-bound DNA) and SDS–PAGE (for the RecN protein). More than 95% of the RecN protein was recovered under all conditions tested (Fig. 1c). The pull-down experiments revealed that His-RecN was able to bind to all three types of DNA with different affinities. Consistent with the results of EMSAs, RecN had a higher affinity for circular ssDNA than circular dsDNA, as determined by the DNA signals in the gel (Fig. 1c, d). There was no obvious difference between the binding of RecN to relaxed and supercoiled circular dsDNA substrates (Supplementary Fig. 1c, d), suggesting that the superhelicity of circular dsDNA does not affect the binding efficiency of RecN. The RecN–circular dsDNA and RecN–circular ssDNA complexes were relatively stable, because increasing concentrations of salt did not reduce the amounts of these complexes (Fig. 1c, d). By contrast, RecN had a low affinity for the linear dsDNA substrate, even in low salt conditions (50 mM), and the DNA–protein complex was abolished by high concentrations of salt (500 mM) (Fig. 1c, d). Taken together, these findings indicate that *E. coli* RecN is a DNA-binding protein with a clear preference for ssDNA, and binds more stably to circular DNA substrates than linear ones.

### Topological DNA-binding activity of RecN

The high-molecular-weight DNA–protein complexes observed in the EMSAs and the high-salt-resistant association to circular DNA could be explained by the formation of intermolecular protein–DNA networks through topological entrapment. To determine whether RecN has topological dsDNA-binding activity, RecN–circular dsDNA complexes were collected using a His pull-down assay and digested with the restriction endonuclease *Pst*I prior to agarose gel electrophoresis (Fig. 1e). The addition of *Pst*I converted circular DNA to linear DNA, which resulted in the release of RecN from the bound DNA (Fig. 1f). A similar result was observed in EMSAs, in which RecN-bound circular dsDNA substrates retained in the well were released into the agarose gel after *Pst*I treatment (Supplementary Fig. 1e). Thus, RecN appears to bind closed circular dsDNA by encircling DNA molecules.

Next, we examined the topological ssDNA-binding activity of RecN in the His pull-down assay, using circular ssDNA annealed to 62-mer complementary oligonucleotides to generate a *Pst*I restriction site (Fig. 1e). Addition of *Pst*I reduced the total amount of RecN-bound ssDNA, although a substantial amount of the complex was still observed in the presence of the restriction endonuclease (Fig. 1g), possibly reflecting the tight association of RecN with ssDNA and/or the inhibition of RecN translocation by secondary structures formed on ssDNA (see the section “Discussion”).

### Intermolecular DNA tethering activity of RecN

**Fig. 2:**
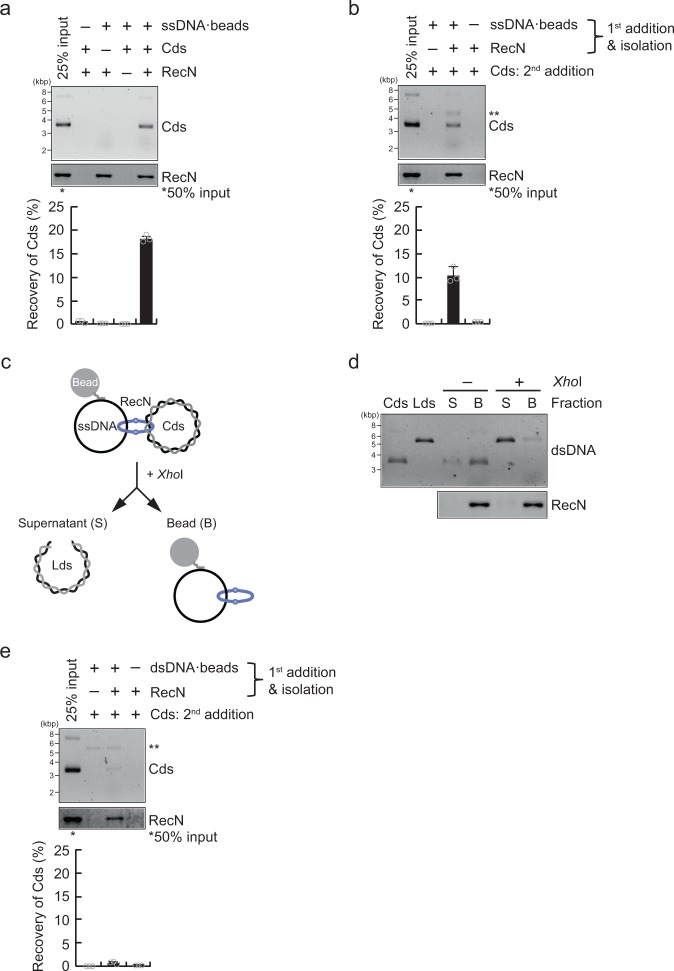
The ssDNA–dsDNA tethering activity of RecN via topological DNA binding. **a** One-step pull-down assays in which RecN and phiX174 circular dsDNA (Cds) were incubated at 37 °C for 10 min in the presence of DNA-free beads or ssDNA beads. The materials bound to the beads were eluted with SDS–sample buffer. The eluted proteins were analyzed by SDS–PAGE and CBB staining, and the eluted Cds was analyzed by agarose gel electrophoresis and SYBR Gold staining. The lower graph shows the intensities of the Cds bands in the agarose gel image. **b** Two-step pull-down assays in which RecN was incubated at 37 °C for 10 min in the presence of DNA-free beads or ssDNA beads. The beads were then washed and incubated with Cds as a second substrate. The bead-bound materials were eluted with SDS–sample buffer and analyzed as described above. The lower graph shows the intensities of the Cds bands in the agarose gel image. **ssDNA dissociated from the beads. **c** Schematic illustration of linear dsDNA (Lds) release by linearization of the captured second Cds in **d**. **d** The ternary complex comprising RecN, dsDNA, and ssDNA beads, isolated by the same method as described for **b** was treated with or without *Xho*I. The bead (B) and the supernatant (S) fractions were analyzed by agarose gel electrophoresis and SDS–PAGE as described above. Cds and Lds are shown as molecular markers. **e** Two-step pull-down assays using dsDNA beads, in which RecN was incubated at 37 °C for 10 min in the presence of DNA-free beads or dsDNA beads. The beads were then washed and incubated with Cds as a secondary substrate. The bead-bound materials were analyzed as described above. The lower graph shows the intensities of Cds bands in the agarose gel image. **dsDNA dissociated from the beads. **a**, **b** and **e** Data represent the mean ± standard deviation of three independent experiments

If RecN supports formation of high molecular weight DNA substrates through its topological DNA-binding activity, it is possible that it interacts simultaneously with at least two different DNA molecules. To examine this possibility, the DNA tethering activity of RecN was analyzed using a pull-down assay with ssDNA beads, which were constructed by annealing a phiX174 circular ssDNA to a complementary biotinylated oligonucleotide immobilized to streptavidin magnetic beads. RecN was incubated with the ssDNA beads and phiX174 circular dsDNA, and then ssDNA bead-bound materials were pulled down and analyzed by agarose gel electrophoresis and SDS–PAGE (Supplementary Fig. 2a). In this “one-step” pull-down assay, the circular dsDNA was specifically recovered in the presence of RecN, but was not recovered in reactions lacking RecN or the ssDNA beads (Fig. 2a). Thus, RecN bound to ssDNA might have an activity to capture other DNA substrates. Similar results were obtained in experiments using M13mp18 circular dsDNA in place of phiX174 circular dsDNA (Supplementary Fig. 3), suggesting that the DNA capture by RecN is independent of sequence homology.

The results described above suggest that RecN loaded onto ssDNA can promote capture of a second DNA molecule. However, if *E. coli* RecN forms multimers, as reported previously for the equivalent protein in *B. subtilis*^34^, its DNA tethering activity could also be explained by an interaction between ssDNA- and dsDNA-bound RecN proteins. To distinguish between these two possibilities, we performed a “two-step” pull-down assay in which RecN was first incubated with ssDNA beads, unbound RecN was washed out with a high-salt buffer, and then circular dsDNA was added to the reaction mixture (Supplementary Fig. 2b). In this experiment, circular dsDNA was efficiently recovered by RecN-bound ssDNA beads, but not by the ssDNA beads alone (Fig. 2b), indicating that the RecN protein loaded onto ssDNA is able to capture a second dsDNA molecule.

Next, we examined whether RecN topologically associates with the second dsDNA molecule. To this end, we treated the ternary complex comprising RecN, dsDNA, and ssDNA beads (generated in the two-step pull-down assay) with the single cut restriction enzyme *Xho*I to linearize the bound circular dsDNA (Fig. 2c). Following digestion, dsDNA was released from the RecN complex (Fig. 2d), suggesting that RecN encircles the second dsDNA molecule. Therefore, RecN might be able to topologically capture two different DNA substrates, namely, circular dsDNA and ssDNA.

The results shown in Fig. 1 indicated that RecN stably and topologically bound to circular dsDNA to a lesser extent than circular ssDNA. Therefore, we investigated whether dsDNA-bound RecN also promotes capture of a second dsDNA molecule. To this end, we performed a two-step pull-down assay with dsDNA beads, in which a dsDNA fragment, biotinylated at both DNA ends, was immobilized on streptavidin magnetic beads (Supplementary Fig. 2b). The amount of the second dsDNA molecule captured by RecN-bound dsDNA beads was markedly lower than that captured by the RecN-ssDNA beads (Fig. 2e), suggesting that initial loading of RecN onto ssDNA, but not dsDNA, is necessary for the secondary dsDNA capture.

### Effect of various nucleotides on the DNA-binding activity of RecN

**Fig. 3:**
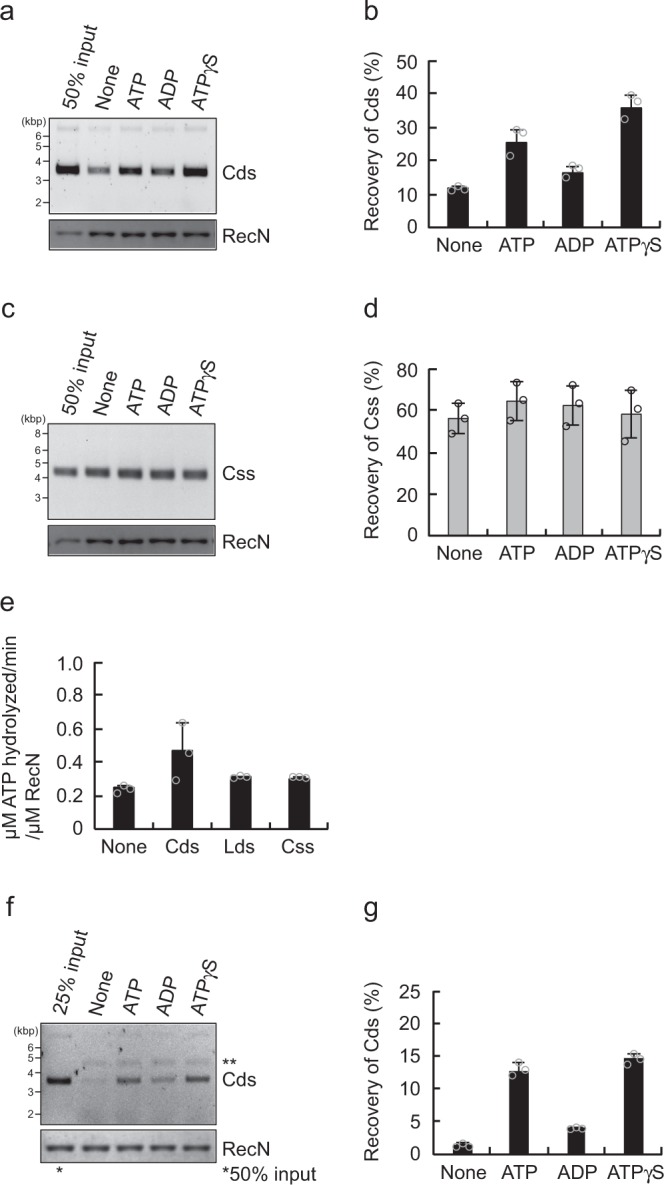
The effect of nucleotides on the DNA-binding activity of RecN. **a** RecN (0.5 µM) was incubated at 37 °C for 10 min in the presence of phiX174 circular dsDNA (Cds) and the indicated nucleotides (2 mM). The RecN–DNA complex was collected using Co^2+^-conjugated beads, washed in buffer containing 500 mM KCl, and eluted with SDS–sample buffer. The eluted proteins and DNA were analyzed as described for Fig. 1c. **b** Quantification of the intensities of the Cds bands in the agarose gel image shown in **a**. **c** The assay was performed as described for **a**, with the exception that phiX174 circular ssDNA (Css) was used in place of Cds. **d** Quantification of the intensities of the Css bands in the agarose gel image shown in **c**. **e** The ATPase activity of RecN. RecN (1 µM) was incubated at 37 °C for 60 min in reaction buffer containing 2 mM ATP plus [γ^32^P]-ATP, in the absence or presence of the indicated phiX174-derivative DNA substrates. The ATPase rates (µM ATP hydrolyzed min^−1^ per µM RecN) were calculated following quantification of the spots of radioactive inorganic phosphate (^32^Pi) using an image analyzer. **f** Two-step pull-down assays in the absence or presence of the indicated nucleotides (2 mM). Cds and RecN recovered by the ssDNA beads were analyzed as described for Fig. 2b. **ssDNA dissociated from the beads. **g** Quantification of the intensities of the Cds bands in the agarose gel image shown in **f**. **b**, **d**, **e**, and **g** All graphs show the mean ± standard deviation of three independent experiments

To investigate the nucleotide dependency of the DNA-binding activity of RecN, we performed a pull-down experiment with His-RecN in the presence of ATP, ADP, or the non-hydrolyzable ATP analog ATP-γ-S. In this experiment, ATP and ATP-γ-S, but not ADP, slightly promoted binding of RecN to circular dsDNA (Fig. 3a, b), whereas none of these nucleotides affected the ability of RecN to bind to circular ssDNA (Fig. 3c, d). These results suggest that ATP binding has some effects on the ability of RecN to bind dsDNA but not ssDNA. RecN was able to hydrolyze ATP, albeit at an extremely low rate (Fig. 3e and Supplementary Fig. 4); however, the addition of various DNA substrates had no obvious effects on the rate of ATP hydrolysis, suggesting that ATP hydrolysis per se is not required for the DNA-binding activity of RecN.

We also examined the effects of ATP, ATP-γ-S, and ADP on the capture of a second dsDNA molecule by RecN using a two-step pull-down assay with ssDNA beads. The nucleotides did not affect formation of the initial RecN–ssDNA complex, as determined by the level of RecN loaded onto ssDNA beads (Fig. 3f). By contrast, ATP and ATP-γ-S, but not ADP, increased the amount of secondary dsDNA captured by RecN (Fig. 3f, g). These results suggest that ATP binding, but not ATP hydrolysis, increases the efficiency of binding of RecN to the second dsDNA molecule.

### RecA prevents dissociation of RecN from ssDNA ends

**Fig. 4:**
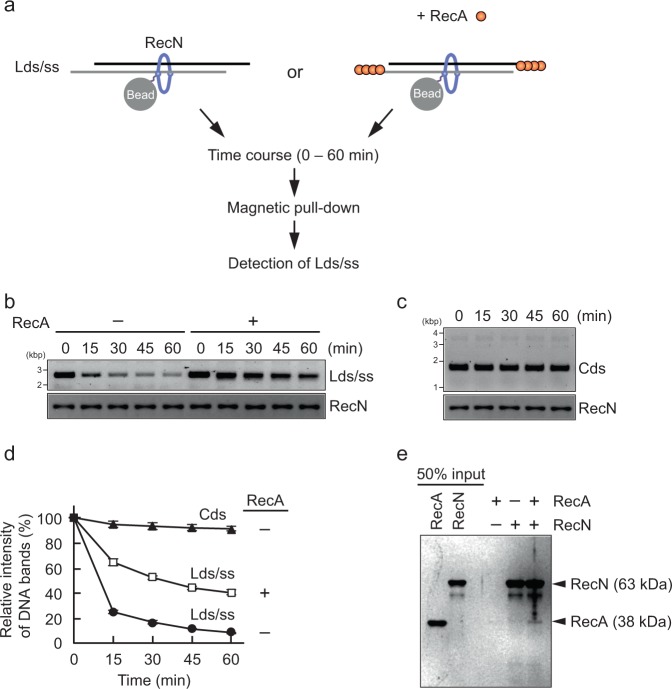
RecA prevents the release of RecN from ssDNA ends. **a** Schematic illustration of His pull-down assays in **b**. **b** RecN (1 µM) was mixed with a linear ds/ssDNA (Lds/ss). The RecN-Lds/ss complex was collected using Co^2+^-conjugated beads, washed in buffer containing 50 mM KCl. To initiate the reactions, the bead suspension was incubated in the presence or absence of RecA (1 µM) at 37 °C. Aliquots were removed for analysis at the indicated time points. The bead-bound materials were recovered and analyzed by agarose gel electrophoresis (for DNA) and SDS–PAGE (for proteins). **c** The control experiment was performed in the absence of RecA as described for **b**, with the exception that pUC19 circular dsDNA (Cds) was used in place of Lds/ss. **d** Quantification of the band intensities of DNA substrates in the agarose gel images shown in **b** and **c**. The amount of DNA recovered at time zero was defined as 100%. Data represent the mean ± standard deviation of three independent experiments. **e** The physical interaction of RecN with RecA. The reaction mixtures were incubated in the presence of the indicated proteins (1 µM each protein). The proteins bound to Co^2+^-conjugated beads were collected, washed, eluted with SDS–sample buffer, and then analyzed by SDS–PAGE and CBB staining

The results described above (Fig. 1) indicated that RecN stably binds circular dsDNA but not linear dsDNA. This finding is explained by the topological association of RecN with DNA; specifically, RecN can slide freely along and therefore dissociate from the ends of linear dsDNA. When DSBs occur in vivo, 3′ ssDNA tails are generated by the RecBCD helicase/nuclease, and RecA is then loaded onto the ssDNA region, resulting in the formation of nucleoprotein filaments. Therefore, we hypothesized that the dissociation of RecN from dsDNA ends might be inhibited by ssDNA regions and/or RecA nucleoprotein filaments. To test this possibility, we constructed a linear DNA substrate containing 3′ ssDNA overhangs of ~300 nt at both ends (linear ds/ssDNA) and examined the effect of RecA on the ability of RecN to bind to this substrate. Linear ds/ssDNA and RecN were incubated and then pulled down using Co^2+^-conjugated magnetic beads. After removing the unbound materials, the resultant complex was incubated with or without RecA at 37 °C. Samples were extracted at different times, and the amounts of the RecN-bound linear ds/ssDNA recovered were analyzed using a His pull-down assay (Fig. 4a). When the reactions were performed in the absence of RecA, the amount of linear ds/ssDNA captured by RecN decreased rapidly in a time-dependent manner (Fig. 4b, d). A control experiment revealed that RecN stably bound circular dsDNA throughout the experiment (Fig. 4c, d). By contrast, the RecN–linear dsDNA complex was unstable and was not recovered in sufficient quantities to perform the experiment (Supplementary Fig. 5a, b). These findings suggest that ssDNA alone is insufficient for stably tethering RecN to linear DNA, although linear ss/dsDNA with ssDNA overhangs, but not linear dsDNA, was substantially retrieved by RecN, despite its linearity (Supplementary Fig. 5a, b). Remarkably, the addition of RecA to the reactions increased the amount of linear ds/ssDNA bound to RecN (Fig. 4b, d). In support of this, the pull-down assay with His-RecN beads revealed that RecN physically interacted with RecA, albeit with weak affinity (Fig. 4e). These results suggest that RecA stabilizes the association between RecN and linear ds/ssDNA by physically and structurally preventing RecN sliding off the DNA ends.

### RecN promotes RecA-mediated D-loop formation

**Fig. 5:**
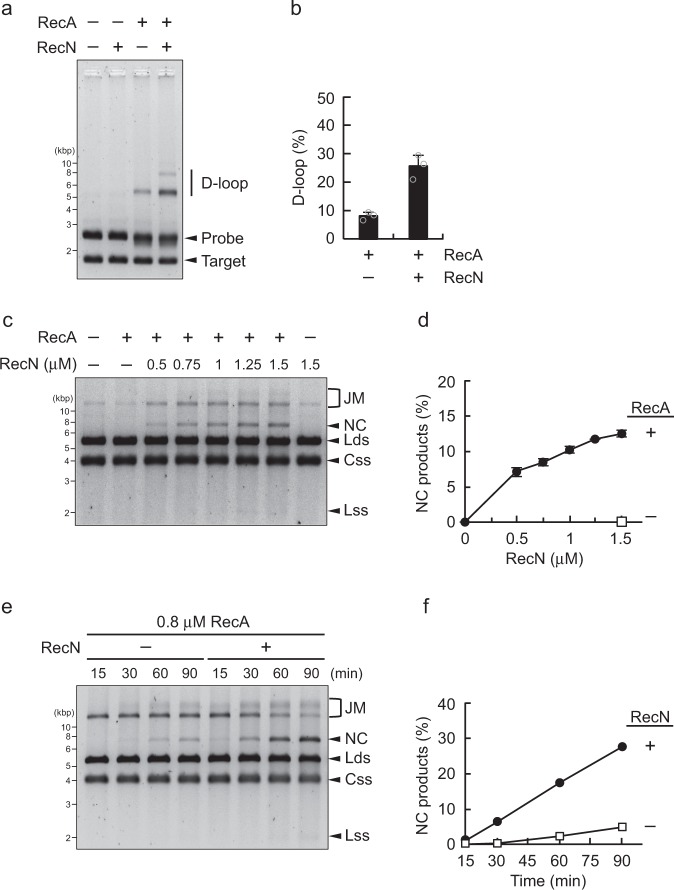
RecN promotes RecA-mediated D-loop formation and DNA strand exchange. **a** D-loop assays. RecA (2 µM) was incubated for 10 min in the presence of the pUC19-derivative linear ds/ssDNA (probe), and then RecN (1 µM) was added to the reaction mixture, followed by incubation for a further 15 min. The reactions were initiated by adding homologous pUC19 supercoiled circular DNA (target) and then incubated for another 5 min. The samples were analyzed by agarose gel electrophoresis and SYBR Gold staining. **b** Quantification of the amounts of D-loop products in the gel image shown in **a**. Data represent the mean ± standard deviation of three independent experiments. **c** Strand exchange assays. RecA (0.6 µM) was incubated for 10 min in the presence of phiX174 circular ssDNA (Css). The indicated concentrations (0–1.5 µM) of RecN were added to the reaction mixture, followed by incubation for 15 min. Subsequently, ssDNA-binding protein was added and samples were incubated for an additional 10 min. The reactions were initiated by adding homologous phiX174 linear dsDNA (Lds), incubated for 90 min, and then stopped by the addition of stop buffer. The samples were analyzed by agarose gel electrophoresis and SYBR Gold staining. **d** Quantification of the amounts of nicked circular dsDNA (NC) products in the gel image shown in **c**. Data represent the mean ± standard deviation of three independent experiments. **e** Time-course strand exchange experiments. RecA (0.8 µM) was incubated with or without RecN (1 µM). Aliquots were collected at the indicated time points and analyzed as described above. **f** Quantification of the amounts of NC DNA products in the gel image shown in **e**. **c**, **e** The arrowheads indicate the DNA substrates (Css and Lds) and the resulting products: joint molecules (JM), nicked circular dsDNA (NC), and linear ssDNA (Lss)

D-loop formation is a critical step in RecA-mediated HR; therefore, we investigated whether RecN promotes this process. In the D-loop assay, we used DNA molecules homologous to both the target dsDNA (negatively supercoiled dsDNA) and linear ds/ssDNA, such that the invading ssDNA region of the linear ds/ssDNA was paired with the complementary strand of the homologous target dsDNA (Supplementary Fig. 6a). When RecA was incubated with these substrates, small amounts of D-loops were produced (Fig. 5a, b and Supplementary Fig. 6b). Notably, the addition of RecN greatly increased the formation of D-loop structures, whereas RecN alone did not promote D-loop formation (Fig. 5a, b). These results suggest that RecN stimulates RecA-mediated D-loop formation.

### RecN promotes RecA-mediated DNA strand exchange

Finally, we examined whether RecN stimulates the RecA-mediated three-strand exchange reaction. In this process, circular ssDNA was first incubated with RecA, and then ssDNA-binding protein was added to the mixture. Finally, the reaction was initiated by the addition of linear dsDNA (Supplementary Fig. 6c). RecA catalyzes the formation of joint molecules by homologous pairing of circular ssDNA with linear dsDNA, and further extensive pairings result in the formation of nicked circular (NC) DNA as a final product (Supplementary Fig. 6d). To assess the effect of RecN on the three-strand exchange reactions, we set up a sub-optimal reaction containing limiting levels of RecA (0.6 µM), such that NC DNA products were barely detected within 90 min of reaction initiation (Fig. 5c, d). The amount of NC DNA produced in the reaction containing 0.6 µM RecA increased in a RecN concentration-dependent manner, whereas RecN alone did not mediate the strand exchange reactions (Fig. 5c, d), suggesting that RecN enhances the strand exchange reaction mediated by RecA. Next, we set up another sub-optimal reaction containing 0.8 µM RecA, in which substantial levels of RecA-mediated joint molecules were detected 15 min after reaction initiation, but NC DNA products were barely detected after 90 min (Fig. 5e, f). Time-course experiments revealed that the addition of RecN increased the amount of NC DNA products formed in the presence of RecA (Fig. 5e, f). Taken together, these results suggest that RecN can not only stimulate the initial RecA-mediated strand invasion, but also the strand exchange reaction itself.

## Discussion

The biochemical analyses performed in this study revealed several structural and functional roles of RecN in HR-dependent DSB repair. Our findings indicate that RecN topologically associates with DNA, RecN loaded onto ssDNA captures a second dsDNA molecule in an ATP-dependent manner, RecN physically interacts with RecA, and RecN stimulates D-loop formation and the strand exchange reaction mediated by RecA. Overall, the data presented here provide mechanistic insights into the recruitment of RecN to DSB sites and sister chromatid interactions, both of which would contribute to the promotion of RecA-mediated HR repair.

The DNA-binding experiments revealed that RecN has topological DNA-binding activity. This activity is a general characteristic of SMC family proteins^37–41^, implying that, like other SMC complexes, the RecN homodimer forms a ring-like structure that embraces DNA within its coiled-coil arms. However, a previous structural study using *D. radiodurans* RecN truncation mutants demonstrated that the RecN homodimer is unlikely to form a closed circular state because it forms a very elongated structure through extensive hydrophobic interactions between the rigid coiled-coil domains of the two monomers^15^. Indeed, bacterial RecN proteins have coiled-coil arms that are less than one-third of the lengths of those of other SMCs; hence it has been assumed that a RecN dimer alone cannot form a ring structure capable of entrapping one or more DNA strands. Previous and this studies suggest that RecN is able to form multimers (larger than tetramers) in solution^15,31,42^. Therefore, we assume that RecN is able to generate ring-like structures by forming tetramers and/or multimers through the head–head engagement between RecN homodimers.

**Fig. 6:**
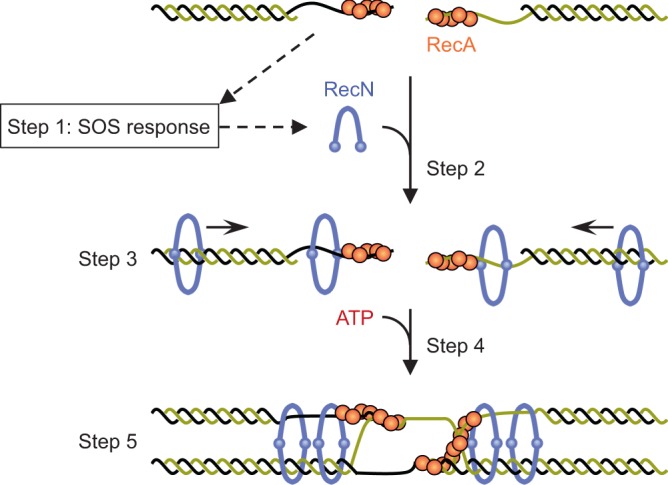
Model for the roles of RecN in DSB repair following DNA end resection. Briefly, RecN expression is stimulated by the SOS response, which is activated after loading of RecA onto ssDNA overhangs (step 1). A RecN multimer (more than or equal to a tetramer) is initially loaded onto ssDNA or dsDNA regions of the damaged strand via topological entrapment (step 2). Subsequently, RecN slides along the DNA and is prevented from disassociating by the RecA filament, resulting in accumulation of RecN molecules at ssDNA regions (step 3). Next, RecN bound to the ssDNA region topologically captures a homologous dsDNA molecule in an ATP-dependent manner (step 4). The resultant ssDNA–dsDNA tethering promotes RecA-mediated homologous pairing and strand exchange (step 5)

RecN may dissociate from the end of linear dsDNA if it is able to slide along the dsDNA freely. This idea is consistent with our findings that RecN-bound linear dsDNA less efficiently than circular dsDNA, and the RecN–circular dsDNA complex was eliminated by restriction enzyme-mediated linearization. Furthermore, we found that RecA reduces dissociation of RecN from linear ds/ssDNA substrates. Together with the observed physical interaction between RecN and RecA, these observations support the notion that RecA nucleoprotein filaments on ssDNA can prevent the dissociation of RecN from the ends of DSBs via a physical interaction and/or serving as structural impediments, thus allowing RecN to translocate to the sites of DSBs (Fig. 6).

Because RecA nucleoprotein filaments are assembled on ssDNA in the 5′–3′ direction, ssDNA gaps could remain in the vicinity of ssDNA/dsDNA junctions^43,44^. In this regard, it is noteworthy that RecN possesses a higher affinity for circular ssDNA than circular dsDNA. We also found that the addition of ATP affected the topological binding of RecN to dsDNA, whereas its ssDNA-binding activity was independent of ATP. Similar behavior has been observed for *E. coli* MukB, a bacterial condensin homolog that displays ATP-independent topological ssDNA-binding activity^45^. Thus, it is likely that RecN uses different modes of binding for ssDNA and dsDNA, the former being ATP-independent and the latter being ATP-dependent, and both activities contribute to its localization at ssDNA regions via different mechanisms (Fig. 6). We speculate that this is related to the importance of oligomerization in one case and not in the other. Binding to dsDNA might require a change in the oligomeric state for topological entrapment and hence needs ATP binding.

We found that RecN loaded onto ssDNA can capture a second dsDNA substrate via topological entrapment. Secondary capture of the dsDNA molecule was markedly stimulated by ATP and ATP-γ-S. By contrast, the RecN–dsDNA complex was barely able to capture a second dsDNA molecule, even in the presence of ATP. These results demonstrate that ssDNA-bound RecN requires ATP binding, but not ATP hydrolysis, to induce the secondary dsDNA tethering activity (Fig. 6). One benefit of this mode of regulation is that RecN cannot capture a second dsDNA until it localizes to DSB sites, thus preventing non-specific DNA–DNA interactions. On the other hand, it remains unclear how RecN mediates specific intermolecular interactions between the damaged strand and its undamaged homologous target. One possibility is that RecN actively tethers two homologous DNA substrates. We consider this scenario unlikely because intermolecular DNA tethering activity is independent of sequence homology in vitro. In addition, a previous study showed that DSB-induced homologous pairing can occur between distant segregated sister loci^46^. An alternative possibility is that RecA-mediated homologous pairing during synapsis might allow RecN to capture the homologous target by promoting proximity between DSB-induced ssDNA and a homologous target. This model is consistent with the previous observations that RecN is required for the preservation of MMC-induced sister chromatid interactions^30^, and that RecA bundles actively facilitate the process of locus pairing between distant sister chromatids^46^. We propose that RecN localized to DSB sites can stably bridge sister chromatids via topological entrapment of homologous targets when RecA-coated ssDNA is arranged in parallel to the homologous dsDNA (Fig. 6).

Previous biochemical studies showed that, once topologically loaded onto DNA, the fission yeast cohesin complex is able to entrap a second DNA, leading to the establishment of sister chromatid cohesion^40,47^. Thus, the biochemical characteristics of RecN would be somewhat similar to those of the cohesin complex, but the order of the reaction differs for these two proteins; specifically, cohesin must initially load onto dsDNA to subsequently capture a ssDNA. Murayama et al. proposed that cohesin is initially recruited to dsDNA on the leading strand and then latches onto ssDNA on the lagging strand^47^, which is a striking contrast to the DNA damage-inducible behaviors of RecN. The opposing orders may reflect the different mechanistic basis for the role of cohesin and RecN.

Although our study focused on understanding how RecN mediates interchromosome interactions between a damaged DNA strand and its homologous target, we do not exclude the possibility that RecN stimulates end-to-end intermolecular interactions of linear DNA, as reported in previous studies of RecN orthologs. Indeed, *E. coli* RecN stimulated the intermolecular ligation of linear dsDNA in the presence of DNA ligase albeit less efficiently (Supplementary Fig. 7). This suggests that in addition to its ssDNA–dsDNA tethering activity, RecN helps tether the two DNA ends generated by a single DSB, and therefore ensures that both DSB ends locate simultaneously to their homologous target.

To date, several biochemical studies of RecN orthologs have shown variable ATPase activities; the ATPase activity of *D. radiodurans* RecN is stimulated in the presence of dsDNA, whereas *B. subtilis* RecN has a ssDNA-specific ATPase activity, and *H. Influenza* RecN has a weak ATPase activity that is not stimulated by DNA^31–33^. A recent study demonstrated that *D. radiodurans* RecN interacts tightly with RecA, and its DNA-dependent ATPase activity is increased by the addition of RecA^36^, suggesting a functional interaction between RecA and RecN, although a role of RecN ATPase activity in promoting RecA-mediated reactions remains unclear. In the current study, the ATPase activity of *E. coli* RecN was very weak and was not stimulated by the addition of DNA. We do not rule out the possibility that stimulation of the ATPase activity of RecN requires additional accessory factors and/or specific conditions that we did not test here. However, it appears that this activity is at least dispensable for binding of RecN to DNA substrates, because ATP-γ-S and ATP had similar effects on the DNA-binding activity of RecN. This is consistent with our previous studies showing that RecNK35A, a RecN Walker A mutant, is recruited to MMC-induced DSBs, but is not disassembled after the release from DNA damage stress^24^. Thus, RecN ATP hydrolysis might be required for the dissociation of RecN from DNA substrates after DSB repair and/or the promotion of RecA-mediated HR reaction. In this regard, it is interesting to note that *E. coli* RecN is rapidly degraded by the ClpXP protease in a manner dependent upon the signaling residues at the C terminus of RecN, which are not conserved among other RecN orthologs^48,49^. Efficient removal of topologically associated RecN and DNA may be essential for the growth recovery process, as these structures could interfere with chromosome segregation and lead to genetic instability^50^. Hence, we speculate that a ClpXP-dependent mechanism may play a role in removing some amount of RecN remaining on the DNA during growth recovery. If this proposal is correct, it might be a plausible explanation for the weak ATPase activity of *E. coli* RecN. Thus, the different mechanisms of RecN disassembly after DSB repair may represent species-specific attributes of ATP hydrolysis.

Our results presented here show that SMC-like RecN is a DNA-binding protein that has a preference for ssDNA and the ability to translocate on dsDNA via topological entrapment. Both of these features are responsible for the localization of RecN at RecA–ssDNA regions. Moreover, RecN loaded onto ssDNA captures a second dsDNA molecule, allowing RecN to embrace two distinct DNA molecules for DSB repair. Our findings support the idea that RecN mediates intermolecular interactions of DNA molecules to promote RecA-dependent DSB repair. Future studies are required to establish whether the *E. coli* RecN-mediated ssDNA–dsDNA tethering function is generally applicable to other RecN orthologs and/or SMC family proteins in DSB repair.

## Methods

### Strain, plasmids, and DNA substrates

An *E. coli* K-12 BW25113 derivative ∆*recN* strain was described previously^48^. *E. coli* genetic and recombinant DNA techniques were performed using standard methods as described previously^51,52^. To construct the overexpression plasmid encoding N-terminally hexahistidine-fused RecN (His-RecN), a fragment containing the *recN* gene derived from the pRecN plasmid^24^ was cloned into pET15b (Novagen), yielding pRecN^OP^. A fragment containing the endogenous SOS promoter and the *recN* gene was cloned into the pSTV28 plasmid (TaKaRa), yielding pSTV28-*recN*. The *recN* gene was then tagged with a hexahistidine cassette at its N-terminus to generate pSTV28-*His·recN*. The construction of recombinant plasmid was confirmed by DNA sequencing. To prepare linear dsDNA, phiX174 supercoiled circular dsDNA (New England Biolabs) was treated with *Pst*I. To generate a *Pst*I restriction site, a mixture of phiX174 circular ssDNA (CssDNA; New England Biolabs) and an oligonucleotide (174del: 5′ GCGTCATGGAAGCGATAAAACTCTGCAGGTTGGATACGCCAATCATTTTTATCGAAGCGCGC 3′) in annealing buffer (50 mM Tris–HCl at pH 7.5, 10 mM MgCl_2_, and 100 mM NaCl) was heated at 60 °C for 30 min and then gradually cooled to 30 °C. Excess oligonucleotides were removed by gel filtration using MicroSpin S-400 HR columns (GE Healthcare). To prepare ssDNA beads, a biotinylated oligonucleotide (Bio174del: 5′ biotin-TGGCTTGAACGCGTCATGGAAGCGATAAAACTCTGCAGGTTGGATACGCCAATCATTTTTATCGAAGCGCGC 3′) was annealed to phiX174 CssDNA as described above, and then the purified biotinylated oligo-annealed CssDNA was immobilized on streptavidin-coated magnetic beads (Dynabeads M-280 Streptavidin; Invitrogen) in Tris–EDTA buffer containing 100 mM KCl and 0.01% NP-40, as described previously^53^. To prepare dsDNA beads, a dsDNA fragment (5.4 kb) biotinylated at both ends was amplified by PCR using a pair of 5′-biotinylated DNA primers (Bio174_2762F: 5′ biotin-TCAAGGACTGTGTGACTATTGACGTCC 3′ and Bio174_2735R: 5′ biotin-CGGTATAATAACCACCATCATGGCGAC 3′) and phiX174 circular dsDNA (5.4 kb) as the template. The fragment was subsequently immobilized on streptavidin-coated magnetic beads as described above. To prepare open circular dsDNA, phiX174 supercoiled circular dsDNA (New England Biolabs) was treated with the Nb. *Bts*I nicking nuclease (New England Biolabs). M13mp18 supercoiled circular dsDNA (M13CdsDNA) was purchased from New England Biolabs.

### Sensitivity tests to MMC

To measure sensitivity to MMC, ∆*recN* cells bearing each of pSTV28 derivatives were exponentially grown at 37 °C in LB medium containing chloramphenicol (10 µg/mL), serially diluted, spotted onto LB plates containing chloramphenicol (20 µg/mL) with or without 0.5 µg/mL MMC, and incubated at 37 °C for 16 h.

### Purification of RecN

The *E. coli* BL21 (DE3) cells carrying a His-RecN expression plasmid derivative of pET15b (Novagen) was grown at 37 °C to an OD_600_ of ~0.6. Isopropyl-β-d-thiogalactopyranoside was added to a final concentration of 1 mM, and the culture was further incubated for 2 h at 37 °C. The cells were collected by centrifugation and the pellet was washed with buffer A (20 mM Tris–HCl at pH 7.5, 150 mM NaCl, and 20% sucrose). The cells were resuspended in buffer B (20 mM Tris–HCl at pH 7.5, 1 M NaCl, 1 mM EDTA, 10% glycerol, 7 mM 2-mercaptoethanol, and 1 mM phenylmethylsulfonyl fluoride), sonicated, and centrifuged at 27,200 × *g* for 20 min. The supernatant was precipitated with ammonium sulfate (30% salt saturation) and centrifuged. The pellet was resuspended in buffer C (20 mM Tris–HCl at pH 7.5, 1 M NaCl, 5% glycerol, and 2 mM 2-mercaptoethanol). The protein was loaded onto a HisTALON Superflow column (Clontech), washed with fifteen column volumes of buffer C containing 10 mM imidazole, and eluted with buffer C containing 500 mM imidazole. The diluted sample was loaded onto a HisTrap FF crude column (GE Healthcare), washed with 10 column volumes of buffer C containing 10 mM imidazole followed by 10 column volumes of buffer C containing 70 mM imidazole, and eluted with a linear gradient of 70–500 mM imidazole over five column volumes. The peak fractions were identified by SDS–PAGE and pooled. The pooled fractions were dialyzed against storage buffer (20 mM Tris–HCl at pH 7.5, 200 mM NaCl, 1 mM EDTA, 50% glycerol, and 1 mM dithiothreitol (DTT)) and stored at −25 °C. We confirmed the absence of nucleic acid contaminations by using spectrophotometry with NanoDrop 8000 (Thermo Scientific).

### Other proteins

*E. coli* RecA was purified as described previously^54^. *E. coli* ssDNA-binding protein (SSB) was purchased from BioAcademia.

### Electrophoretic mobility shift assay (EMSA)

RecN (0–1.5 µM) was incubated at 37 °C for 10 min in 5 µL buffer E (20 mM Tris–HCl at pH 7.5, 0.01% NP-40, and 1 mM DTT) containing 50 mM KCl, 2 mM MgCl_2_, 5% glycerol, and 2 mM ATP in the presence of 1.5 nM phiX174-derivative DNA substrates (as a circular or linear molecule). To linearize circular dsDNA, the reaction mixtures were further incubated at 37 °C for 15 min in the presence of *Pst*I (10 units). Samples were separated by 0.8% agarose gel electrophoresis with 0.5 × TBE buffer and then stained with SYBR Gold (Invitrogen). Protein-free DNA was quantified using ImageJ software (NIH) and presented as a percentile.

### Pull-down assay to assess binding of RecN to DNA substrates

RecN (0.5 µM) was incubated at 37 °C for 10 min in 10 µL buffer D (20 mM Tris–HCl at pH 7.5, 0.01% NP-40, and 7 mM 2-mercaptoethanol) containing 50 mM KCl, 2 mM MgCl_2_, 5% glycerol, and 2 mM ATP (ADP or ATP-γ-S as necessary) in the presence of 1.5 nM phiX174-derivative DNA substrates (as a circular or linear molecule). The mixture was further incubated at 4 °C for 15 min with gentle rotation in the presence of Co^2+^-conjugated magnetic beads (1 µL suspension; Invitrogen) equilibrated in buffer D containing 50 mM KCl. The beads were collected and washed with buffer D containing 2 mM MgCl_2_, 20 mM imidazole, and 50 or 500 mM KCl. Proteins and DNA were eluted from the beads using SDS–sample buffer I (43 mM Tris–HCl at pH 6.8, 1.4% SDS, 14% glycerol, 20 mM EDTA, 540 mM 2-mercaptoethanol, and 0.04% bromophenol blue). Proteins were analyzed by 10% SDS–PAGE and Coomassie Brilliant Blue (CBB) staining. DNA was analyzed by 0.8% agarose gel electrophoresis with 1 × TAE buffer and SYBR Gold staining. The intensities of the DNA bands or the protein bands were quantified using ImageJ software. To linearize RecN-bound circular DNA, the RecN–DNA complex was collected using magnetic beads as described above. The bead-bound complex was incubated at 37 °C for 30 min in 10 µL buffer D containing 100 mM KCl, 3 mM MgCl_2_, and 20 mM imidazole in the absence or presence of *Pst*I (20 units). The salt concentration was adjusted to 500 mM NaCl, and then the first supernatant fraction (S1) was recovered. The beads were resuspended in buffer D containing 500 mM NaCl and 2 mM MgCl_2_, and then the second supernatant fraction (S2) was recovered. The bead fraction and the total supernatant fraction obtained by mixing S1 and S2 were analyzed by 10% SDS–PAGE and CBB staining (for proteins), and by 0.8% agarose gel electrophoresis with 1 × TAE buffer or 0.5 × TBE buffer and SYBR Gold staining (for DNA).

### One-step pull-down assay

Simple methods are presented in Supplementary Fig. 2a. Specifically, RecN (1 µM) was incubated at 37 °C for 10 min in 10 µL buffer E containing 100 mM KCl, 2 mM MgCl_2_, 2 mM ATP (ADP or ATP-γ-S as necessary), and 5% glycerol in the presence of 0.75 nM phiX174 circular dsDNA (as a DNA molecule) and phiX174 DNA beads (containing 0.75 nM DNA molecules). The beads were collected and washed with buffer E containing 1 M KCl and 2 mM MgCl_2_. The bound proteins and DNA were eluted from the beads using SDS–sample buffer II (22 mM Tris–HCl at pH 6.8, 0.7% SDS, 7% glycerol, 270 mM 2-mercaptoethanol, and 0.02% bromophenol blue). Proteins were analyzed by 10% SDS–PAGE and CBB staining. DNA was analyzed by 0.8% agarose gel electrophoresis with 1 × TAE buffer and SYBR Gold staining. DNA band intensities were quantified using ImageJ software.

### Two-step pull-down assay

Simple methods are presented in Supplementary Fig. 2b. Specifically, RecN–DNA complexes on the beads were isolated using the same method as described for the one-step pull-down assay, with the exception that circular dsDNA was omitted from the reaction mixtures. The isolated complexes were incubated at 37 °C for 10 min in 10 µL buffer E containing 50 mM KCl, 2 mM MgCl_2_, 2 mM ATP (ADP or ATP-γ-S as necessary), and 5% glycerol in the presence of 0.75 nM phiX174 circular dsDNA (as a DNA molecule) as a second substrate. The bead-bound materials were analyzed as described in the one-step pull-down assay. To linearize circular dsDNA bound to RecN on ssDNA beads, the ternary complex was isolated by the same method as described above. The complex was incubated at 37 °C for 30 min in 10 µL buffer E containing 50 mM KCl and 3 mM MgCl_2_ in the absence or presence of *Xho*I (10 units). The salt concentration was adjusted to 500 mM NaCl, and then the first supernatant fraction (S1) was recovered. The beads were resuspended in buffer E containing 500 mM NaCl and 2 mM MgCl_2,_ and then the second supernatant fraction (S2) was recovered. The bead fraction and the total supernatant fraction obtained by mixing S1 and S2 were analyzed by 10% SDS–PAGE and CBB staining (for the proteins), and by 0.8% agarose gel electrophoresis with 1 × TAE buffer and SYBR Gold staining (for DNA).

### ATPase assay

RecN (1 µM) was incubated at 37 °C in buffer E containing 50 mM NaCl, 2 mM MgCl_2_, 5% glycerol, and 2 mM ATP plus [γ^32^P]-ATP (PerkinElmer) in the absence or presence of 1.5 nM phiX174-derivative DNA substrates (as a circular or linear molecule). Reaction aliquots were collected at 0, 15, 30, and 60 min, and added to an equal volume of stop buffer (100 mM Tris–HCl at pH 7.5, 100 mM EDTA, and 20 mM ADP). ATP and inorganic phosphate (Pi) in the samples were separated by thin-layer chromatography and quantified using a BAS2500 imaging analyzer (Fujifilm).

### Linear ds/ssDNA-binding activity of RecN

To prepare a linear dsDNA substrate containing 3′ ssDNA tails (Lds/ssDNA), a linear pUC19 dsDNA digested with *Ssp*I-HF (New England Biolabs) was treated with T7 exonuclease (5′–3′ exonuclease) at 25 °C for 10 s, yielding linear ds/ssDNA containing a 3′-ssDNA end with an average length of 300 bases. RecN (1 µM) was incubated at 37 °C for 10 min in 5 µL buffer D containing 50 mM KCl, 2 mM MgCl_2_, 5% glycerol, and 2 mM ATP in the presence of 3 nM Lds/ssDNA (as a linear molecule). The RecN–Lds/ssDNA complex was isolated using Co^2+^-conjugated beads and washed with buffer D containing 2 mM MgCl_2_, 20 mM imidazole, and 50 mM KCl. The bead-bound complex was incubated at 37 °C in 10 µL buffer D containing 150 mM KCl, 3 mM MgCl_2_, 5% glycerol, 20 mM imidazole, 3 mM ATP, 8 mM creatine phosphate (CP), and 8 units/mL creatine kinase (CK) in the absence or presence of 1 µM RecA. The reaction mixtures were harvested at the indicated time points, and the beads were collected and washed with buffer D containing 50 mM KCl, 2 mM MgCl_2_, and 20 mM imidazole. Proteins and DNA were eluted with SDS–sample buffer I and analyzed by 10% SDS–PAGE and CBB staining (for proteins), and by 0.8% agarose gel electrophoresis with 1 × TAE buffer and SYBR Gold staining (for DNA). DNA band intensities were quantified using ImageJ software.

### Pull-down assay to assess binding of RecN to RecA

RecN (1 µM) was incubated at 37 °C for 10 min in 10 µL buffer D (20 mM Tris–HCl at pH 7.5, 0.01% NP-40 and 7 mM 2-mercaptoethanol) containing 50 mM KCl, 2 mM MgCl_2_, 5% glycerol, and 2 mM ATP in the presence of RecA (1 µM). The mixture was further incubated at 4 °C for 15 min with gentle rotation in the presence of 10 mM imidazole and Co^2+^-conjugated magnetic beads (1 µL suspension) equilibrated in buffer D containing 50 mM KCl. The beads were collected and washed with buffer D containing 100 mM KCl, 2 mM MgCl_2_, and 20 mM imidazole. Proteins were eluted from the beads using SDS–sample buffer I, and analyzed by 10% SDS–PAGE and CBB staining.

### D-loop assay

A modified version of a previously reported method^36^ was used. In this assay, RecA (2 µM) was incubated for 10 min on ice in 10 µL buffer SE (33 mM Tris–HCl at pH 7.5, 40 mM NaCl, 10 mM MgCl_2_, 1 mM DTT, 12% glycerol, 3 mM ATP, and an ATP-regeneration system (8 mM CP and 8 units/mL CK)) containing Lds/ssDNA (10 µM as a nucleotide), and then RecN (1 µM) was added and samples were incubated at 37 °C for 15 min. Reactions were initiated by adding pUC19 supercoiled circular dsDNA (10 µM as a nucleotide) and then samples were incubated for 5 min. Reactions were stopped by adding stop buffer (0.8% SDS and 0.54 mg/mL proteinase K). Samples were analyzed by 0.8% agarose gel electrophoresis with 1 × TAE buffer and SYBR Gold staining. DNA products were quantified using ImageJ software.

### DNA strand exchange assay

The RecA-mediated three-strand exchange reaction was performed as described previously^55^. Briefly, the indicated concentrations of RecA were incubated for 10 min on ice in 10 µL buffer SE in the presence of 10 µM phiX174 circular ssDNA (as a nucleotide), and then the indicated concentrations of RecN were added and samples were incubated at 37 °C for 15 min. SSB (2 µM) was added and samples were further incubated for 10 min. Reactions were initiated by adding phiX174 linear dsDNA (10 µM as a nucleotide) and then samples were incubated for 90 min or the indicated durations. Reactions were stopped by adding the stop buffer. Samples were analyzed as described for the D-loop assay.

### DNA ligation assay

This assay was essentially carried out as described previously^32^. Briefly, the indicated concentrations of RecN were incubated at 37 °C for 30 min in 50 µL buffer ED (25 mM Tris–HCl at pH 7.5, 40 mM KOAc, 17.5 mM Mg (OAc)_2_, 1 mM DTT, 2.5% PEG 8000, 3 mM ATP, 4 mM phosphoenol pyruvate, and 7 units/mL pyruvate kinase) containing 2.4 nM blunt-ended linear dsDNA (as a linear molecule) which is derived from pUC19 (2.7 kb) digested with *Ssp*I-HF (New England Biolabs). The samples were further incubated at 22 °C for 30 min after adding 5.5 µL of 10 × DNA ligase buffer (Thermo scientific) and 5 units of T4 DNA ligase (Thermo scientific). The DNA in each sample was subsequently recovered by ethanol precipitation, resuspended in 10 µL of TE buffer containing 1 × loading buffer (TaKaRa) and analyzed by 0.5% agarose gel electrophoresis with 1 × TAE buffer and ethidium bromide staining.

### Quantification and statistical analysis

DNA samples were separated using agarose gel electrophoresis. After staining with SYBR Gold, the DNA bands were detected using Gel Doc EZ imager (Bio-Rad) with Image Lab software (Bio-Rad), and its intensities were quantified using ImageJ software (NIH) as a percentage of DNA input, loaded alongside. Protein samples were separated using SDS–PAGE. After staining with CBB, the proteins were detected using Gel Doc EZ imager with Image Lab software, and quantified using ImageJ software as a percentage of protein input, loaded alongside. In ATPase assays, reaction products were separated by thin-layer chromatography and quantified using a BAS2500 imaging analyzer (Fujifilm) with ImageReader software (Fujifilm) and ImageGauge software (Fujifilm). ATP hydrolysis at each time point (0, 15, 30, and 60 min) was calculated from the ratio of inorganic monophosphate and ATP, and the ATPase rates (µM ATP hydrolyzed min^−1^ per µM RecN) were calculated from the obtained values. The graphs essentially represent the mean ± standard deviation of three independent experiments as indicated in corresponding figure legends.

### Reporting summary

Further information on research design is available in the Nature Research Reporting Summary linked to this article.

## Supplementary information

Supplementary Information

Reporting Summary

## Data Availability

The data supporting the findings of this study are available within this published article and its Supplementary information file. The source data reported in this study has been deposited in Dryad repository^56^. Full gels are shown in Supplementary Information.

## References

[1] Cromie GA, Connelly JC, Leach DR (2001). Recombination at double-strand breaks and DNA ends: conserved mechanisms from phage to humans. Mol. Cell.

[2] Wyman C, Ristic D, Kanaar R (2004). Homologous recombination-mediated double-strand break repair. DNA Repair.

[3] Amundsen SK, Smith GR (2003). Interchangeable parts of the *Escherichia coli* recombination machinery. Cell.

[4] Kowalczykowski SC (2000). Initiation of genetic recombination and recombination-dependent replication. Trends Biochem. Sci..

[5] Singleton MR, Dillingham MS, Gaudier M, Kowalczykowski SC, Wigley DB (2004). Crystal structure of RecBCD enzyme reveals a machine for processing DNA breaks. Nature.

[6] Cox MM, Lehman IR (1987). Enzymes of general recombination. Annu. Rev. Biochem..

[7] Courcelle J, Khodursky A, Peter B, Brown PO, Hanawalt PC (2001). Comparative gene expression profiles following UV exposure in wild-type and SOS-deficient *Escherichia coli*. Genetics.

[8] Little JW (1991). Mechanism of specific LexA cleavage: autodigestion and the role of RecA coprotease. Biochimie.

[9] Little JW, Mount DW (1982). The SOS regulatory system of *Escherichia coli*. Cell.

[10] Luo Y (2001). Crystal structure of LexA: a conformational switch for regulation of self-cleavage. Cell.

[11] Finch PW, Chambers P, Emmerson PT (1985). Identification of the *Escherichia coli* recN gene product as a major SOS protein. J. Bacteriol..

[12] Graumann PL, Knust T (2009). Dynamics of the bacterial SMC complex and SMC-like proteins involved in DNA repair. Chromosome Res..

[13] Hirano T (2006). At the heart of the chromosome: SMC proteins in action. Nat. Rev. Mol. Cell Biol..

[14] Lloyd RG, Picksley SM, Prescott C (1983). Inducible expression of a gene specific to the RecF pathway for recombination in *Escherichia coli* K12. Mol. Gen. Genet..

[15] Pellegrino S (2012). Structural and functional characterization of an SMC-like protein RecN: new insights into double-strand break repair. Structure.

[16] Rostas K, Morton SJ, Picksley SM, Lloyd RG (1987). Nucleotide sequence and LexA regulation of the *Escherichia coli* recN gene. Nucleic Acids Res..

[17] Hirano T (2016). Condensin-based chromosome organization from bacteria to vertebrates. Cell.

[18] Jeppsson K, Kanno T, Shirahige K, Sjögren C (2014). The maintenance of chromosome structure: positioning and functioning of SMC complexes. Nat. Rev. Mol. Cell Biol..

[19] Nasmyth K (2011). Cohesin: a catenase with separate entry and exit gates?. Nat. Cell Biol..

[20] Oravcová, M. & Boddy, M. N. Recruitment, loading, and activation of the Smc5–Smc6 SUMO ligase. *Curr. Genet*. 10.1007//s00294-018-0922-9 (2019). 10.1007/s00294-018-0922-9 PMC6511331 30600397

[21] Peters JM, Nishiyama T (2012). Sister chromatid cohesion. Cold Spring Harb. Perspect. Biol..

[22] Thadani R, Uhlmann F, Heeger S (2012). Condensin, chromatin crossbarring and chromosome condensation. Curr. Biol..

[23] Uhlmann F (2016). SMC complexes: from DNA to chromosomes. Nat. Rev. Mol. Cell Biol..

[24] Keyamura K, Sakaguchi C, Kubota Y, Niki H, Hishida T (2013). RecA protein recruits structural maintenance of chromosomes (SMC)-like RecN protein to DNA double-strand breaks. J. Biol. Chem..

[25] Kosa JL, Zdraveski ZZ, Currier S, Marinus MG, Essigmann JM (2004). RecN and RecG are required for *Escherichia coli* survival of Bleomycin-induced damage. Mutat. Res..

[26] Meddows TR, Savory AP, Grove JI, Moore T, Lloyd RG (2005). RecN protein and transcription factor DksA combine to promote faithful recombinational repair of DNA double-strand breaks. Mol. Microbiol..

[27] Picksley SM, Attfield PV, Lloyd RG (1984). Repair of DNA double-strand breaks in *Escherichia coli* K12 requires a functional recN product. Mol. Gen. Genet..

[28] Sargentini NJ, Smith KC (1983). Characterization of an *Escherichia coli* mutant (radB101) sensitive to gamma and UV radiation, and methyl methanesulfonate. Radiat. Res..

[29] Sargentini NJ, Smith KC (1986). Quantitation of the involvement of the recA, recB, recC, recF, recJ, recN, lexA, radA, radB, uvrD, and umuC genes in the repair of X-ray-induced DNA double-strand breaks in *Escherichia coli*. Radiat. Res..

[30] Vickridge E, Planchenault C, Cockram C, Junceda IG, Espéli O (2017). Management of *E. coli* sister chromatid cohesion in response to genotoxic stress. Nat. Commun..

[31] Grove JI, Wood SR, Briggs GS, Oldham NJ, Lloyd RG (2009). A soluble RecN homologue provides means for biochemical and genetic analysis of DNA double-strand break repair in *Escherichia coli*. DNA Repair.

[32] Reyes ED, Patidar PL, Uranga LA, Bortoletto AS, Lusetti SL (2010). RecN is a cohesin-like protein that stimulates intermolecular DNA interactions in vitro. J. Biol. Chem..

[33] Sanchez H, Alonso JC (2005). Bacillus subtilis RecN binds and protects 3’-single-stranded DNA extensions in the presence of ATP. Nucleic Acids Res..

[34] Sanchez H, Cardenas PP, Yoshimura SH, Takeyasu K, Alonso JC (2008). Dynamic structures of *Bacillus subtilis* RecN–DNA complexes. Nucleic Acids Res..

[35] Losada A, Hirano T (2001). Intermolecular DNA interactions stimulated by the cohesin complex in vitro: implications for sister chromatid cohesion. Curr. Biol..

[36] Uranga LA, Reyes ED, Patidar PL, Redman LN, Lusetti SL (2017). The cohesin-like RecN protein stimulates RecA-mediated recombinational repair of DNA double-strand breaks. Nat. Commun..

[37] Cuylen S, Metz J, Haering CH (2011). Condensin structures chromosomal DNA through topological links. Nat. Struct. Mol. Biol..

[38] Haering CH, Farcas AM, Arumugam P, Metson J, Nasmyth K (2008). The cohesin ring concatenates sister DNA molecules. Nature.

[39] Kanno T, Berta DG, Sjögren C (2015). The Smc5/6 Complex Is an ATP-Dependent Intermolecular DNA Linker. Cell Rep..

[40] Murayama Y, Uhlmann F (2014). Biochemical reconstitution of topological DNA binding by the cohesin ring. Nature.

[41] Wilhelm L (2015). SMC condensin entraps chromosomal DNA by an ATP hydrolysis dependent loading mechanism in *Bacillus subtilis*. Elife.

[42] Kidane D, Sanchez H, Alonso JC, Graumann PL (2004). Visualization of DNA double-strand break repair in live bacteria reveals dynamic recruitment of *Bacillus subtilis* RecF, RecO and RecN proteins to distinct sites on the nucleoids. Mol. Microbiol..

[43] Joo C (2006). Real-time observation of RecA filament dynamics with single monomer resolution. Cell.

[44] Register JC, Griffith J (1985). The direction of RecA protein assembly onto single strand DNA is the same as the direction of strand assimilation during strand exchange. J. Biol. Chem..

[45] Niki H, Yano K (2016). In vitro topological loading of bacterial condensin MukB on DNA, preferentially single-stranded DNA rather than double-stranded DNA. Sci. Rep..

[46] Lesterlin C, Ball G, Schermelleh L, Sherratt DJ (2014). RecA bundles mediate homology pairing between distant sisters during DNA break repair. Nature.

[47] Murayama Y, Samora CP, Kurokawa Y, Iwasaki H, Uhlmann F (2018). Establishment of DNA–DNA interactions by the cohesin ring. Cell.

[48] Nagashima K (2006). Degradation of *Escherichia coli* RecN aggregates by ClpXP protease and its implications for DNA damage tolerance. J. Biol. Chem..

[49] Neher SB (2006). Proteomic profiling of ClpXP substrates after DNA damage reveals extensive instability within SOS regulon. Mol. Cell.

[50] Warr AR, Klimova AN, Nwaobasi AN, Sandler SJ, Protease-deficient SOS (2018). constitutive cells have RecN-dependent cell division phenotypes. Mol. Microbiol.

[51] Miller, J. H. *A Short Course in Bacterial Genetics. A Laboratory Manual and Handbook for Escherichia coli and Related Bacteria* (Cold Spring Harber Laboratory Press, Cold Spring Harbor, NY, 1992).

[52] Sambrook, J., Fritsch, E. F. & Maniatis, T. *Molecular Cloning. A Laboratory Manual*, 2nd edn (Cold Spring Harbor Laboratory Press, Cold Spring Harbor, NY, 1989).

[53] Kurokawa Y, Murayama Y, Haruta-Takahashi N, Urabe I, Iwasaki H (2008). Reconstitution of DNA strand exchange mediated by Rhp51 recombinase and two mediators. PLoS Biol..

[54] Hishida T, Iwasaki H, Han YW, Ohnishi T, Shinagawa H (2003). Uncoupling of the ATPase activity from the branch migration activity of RuvAB protein complexes containing both wild-type and ATPase-defective RuvB proteins. Genes Cells.

[55] Haruta N (2006). The Swi5–Sfr1 complex stimulates Rhp51/Rad51- and Dmc1-mediated DNA strand exchange in vitro. Nat. Struct. Mol. Biol..

[56] Keyamura, N. & Hishida, T. Data from: topological DNA-binding of structural maintenance of chromosomes-like RecN promotes DNA double-strand break repair in *E. coli*. *Dryad Digit. Repos*. 10.5061/dryad.7p3p8j3 (2019). 10.1038/s42003-019-0655-4 PMC6856136 31754643

