# Supplementary material for: Topological DNA-binding of structural maintenance of chromosomes-like RecN promotes DNA double-strand break repair in Escherichia coli
Source: Commun Biol. 2019 Nov 14;2:413. doi: 10.1038/s42003-019-0655-4 (PMC6856136; doi:10.1038/s42003-019-0655-4)
Supplement: Supplementary file 1 — Supplementary Information [file 42003_2019_655_MOESM1_ESM.pdf]

Supplementary Figure 1

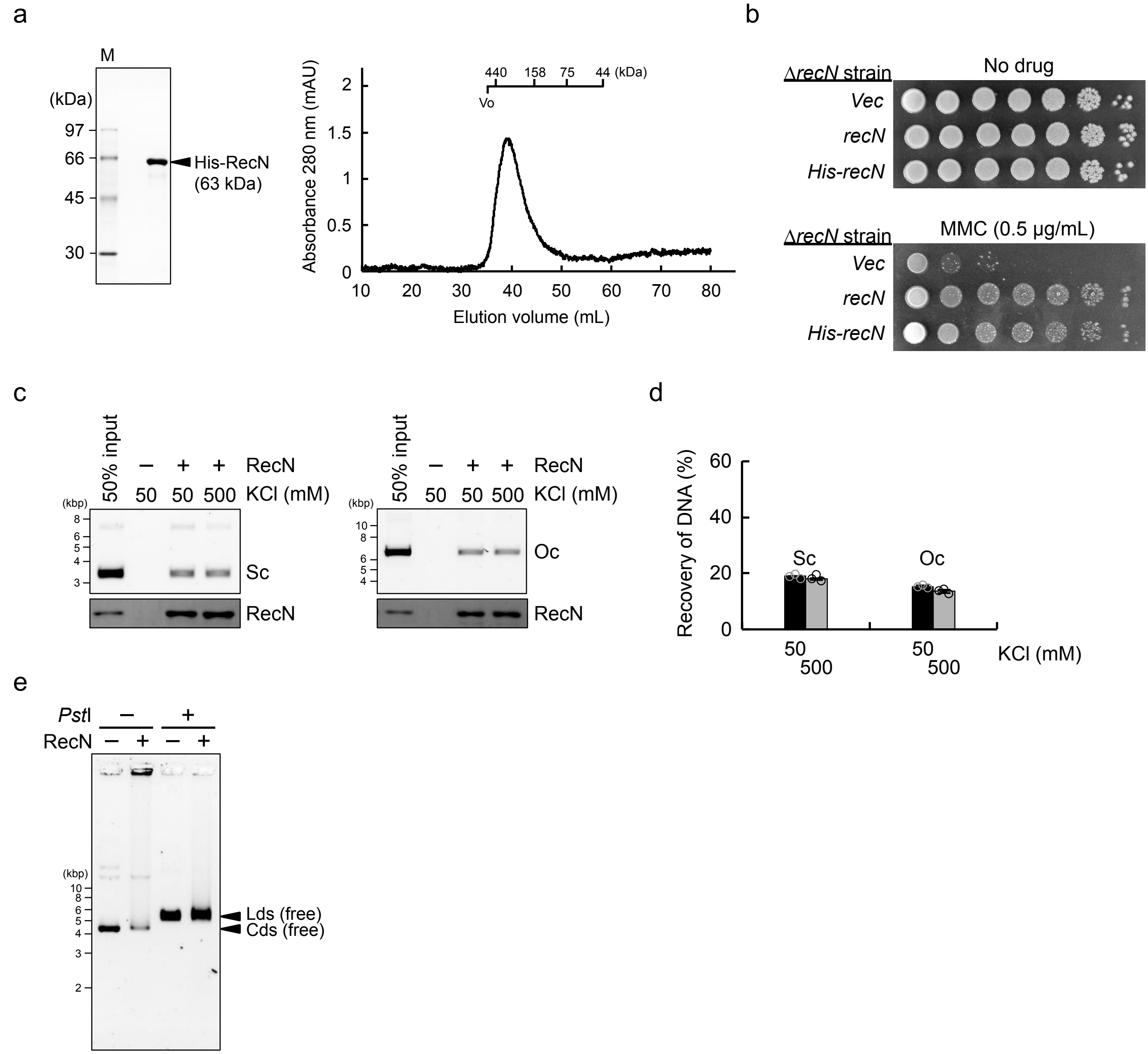

Supplementary Figure 1 Profiling of the circular dsDNA-binding activity of RecN.

a, The purified hexahistidine-tagged RecN (His-RecN; 63 kDa) protein (1 μg) was stained with Coomassie Brilliant Blue (CBB) after 10% SDS-PAGE (left). M, molecular size marker. The protein was also analyzed using gel filtration chromatography with a HiPrep 16/60 Sephacryl S-200 HR column (right). The positions of the void (Vo) volume (Blue dextran (2,000 kDa)) and molecular size markers (Ferritin (440 kDa), Aldolase (158 kDa), Conalbumin (75 kDa), and Ovalbumin (44 kDa)) were determined under the same conditions. **b,** Dilutions of Δ*recN* cells carrying pSTV28 (vector) or pSTV28 derivatives encoding the SOS-inducible wild-type RecN (*recN*) or His-RecN (*His-recN*) were spotted onto LB plus chloramphenicol plates, with or without MMC (0.5 μg/mL), and incubated at 37°C. **c,** Comparison of the ability of RecN to bind to phiX174 supercoiled circular DNA (Sc) and phiX174 relaxed open circular DNA (Oc). The assays were carried out using the same method as described for Fig. 1c. **d,** Quantification of the band intensities of DNA substrates in the agarose gel images shown in **c**. Data are shown as the mean ± standard deviation of three independent experiments. **e,** Electrophoretic mobility shift assays in which RecN (0 or 1.5 μM) was incubated at 37˚C for 10 min in the presence of phiX174 circular dsDNA (Cds) and then incubated with or without the single cut restriction enzyme *Pst*I which generated linear dsDNA (Lds). The samples were analyzed by agarose gel electrophoresis and SYBR Gold staining.

Supplementary Figure 2

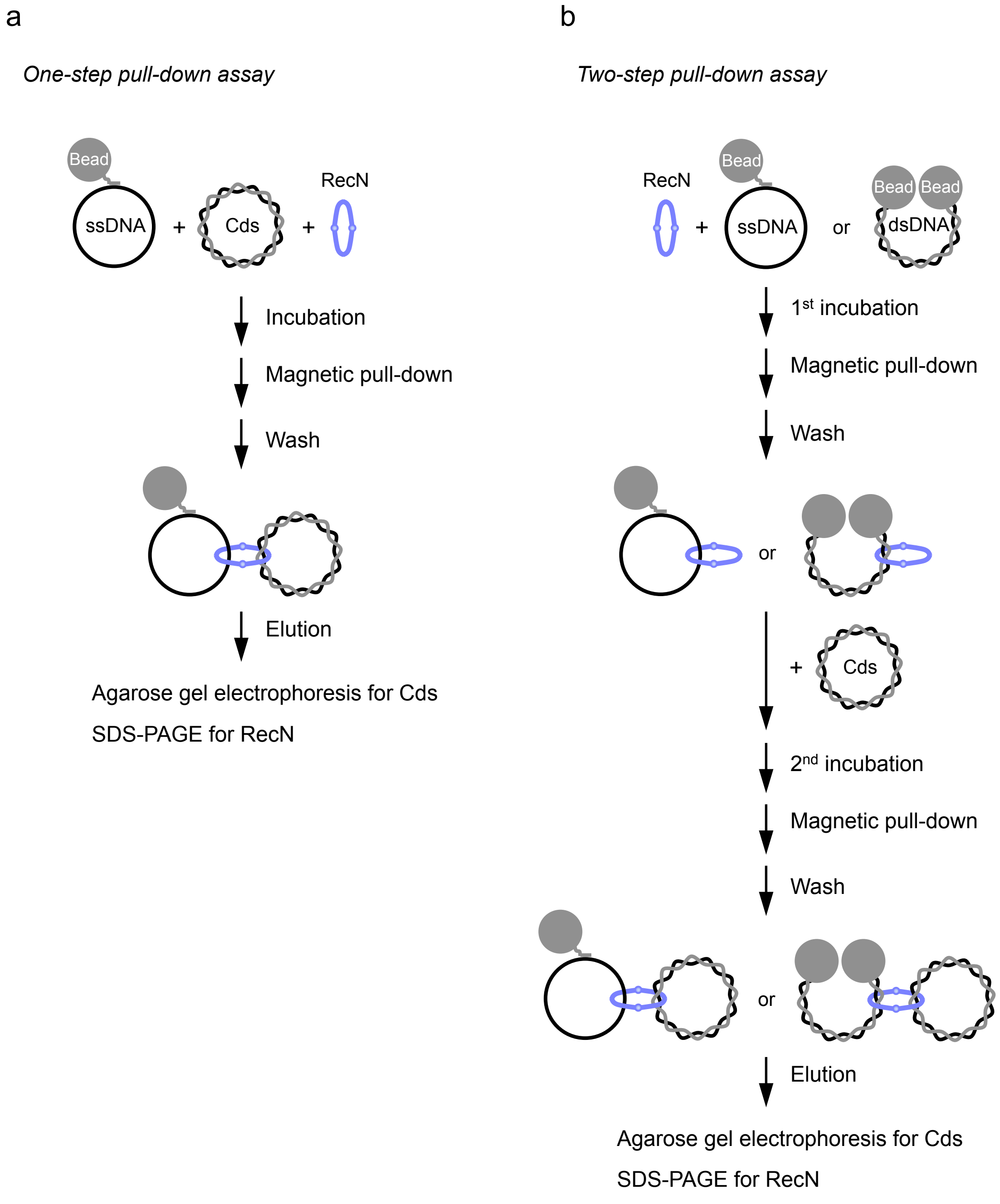

Supplementary Figure 2 One-step and two-step pull-down assays using DNA-conjugated magnetic beads. Overview of the one-step (**a**) and two-step (**b**) pull-down assays used to examine the DNA-DNA tethering activities of RecN (see Methods for more details).

Supplementary Figure 3

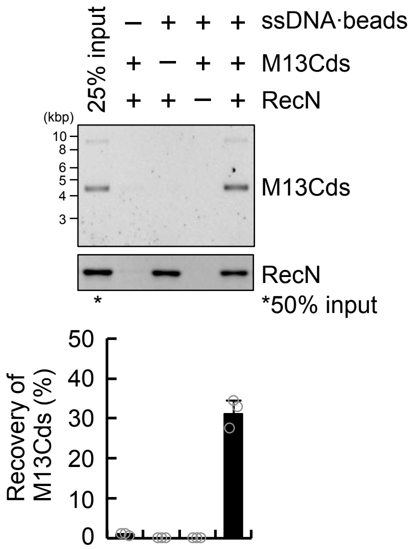

Supplementary Figure 3 Pull-down in the presence of M13 dsDNA, ssDNA (phiX174) beads, and RecN. One-step pull-down assays were carried out essentially as described in Figure 2a, except that M13mp18 circular dsDNA (M13Cds) was used instead of phiX174 circular dsDNA. RecN and M13Cds were incubated at 37°C for 10 min in the presence of DNA-free beads or ssDNA (phiX174 derivatives) beads. The materials bound to the beads were eluted with SDS-sample buffer. The eluted proteins were analyzed by SDS-PAGE and CBB staining, and the eluted M13Cds was analyzed by agarose gel electrophoresis and SYBR Gold staining. The lower graph shows the intensities of the M13Cds bands in the agarose gel image. Data represent the mean ± standard deviation of three independent experiments.

Supplementary Figure 4

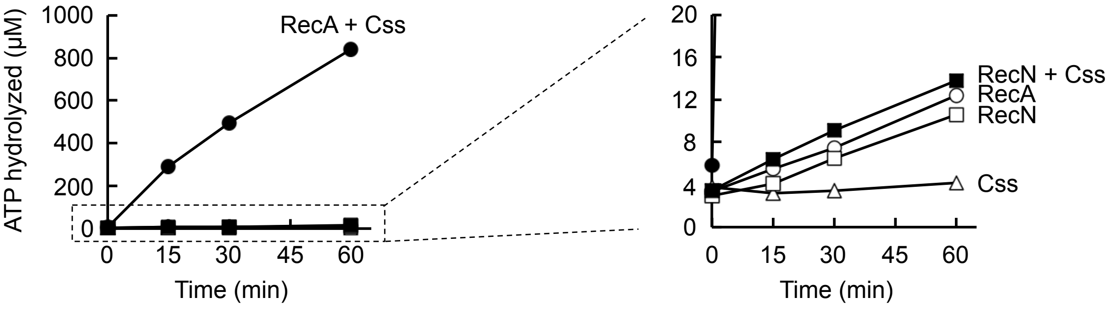

Supplementary Figure 4 The ATPase activity of RecN.
RecN (1 μM) was incubated at 37°C for the indicated times in reaction buffer containing 2 mM ATP plus [γ^{32}P]-ATP, in the absence or presence of phiX174 circular ssDNA (Css). The amounts of ATP hydrolyzed were calculated after quantification of the spots of radioactive inorganic phosphate (^{32}Pi) using an imaging analyzer. As a control, the ATPase activity of RecA (1 μM) was also examined.

Supplementary Figure 5

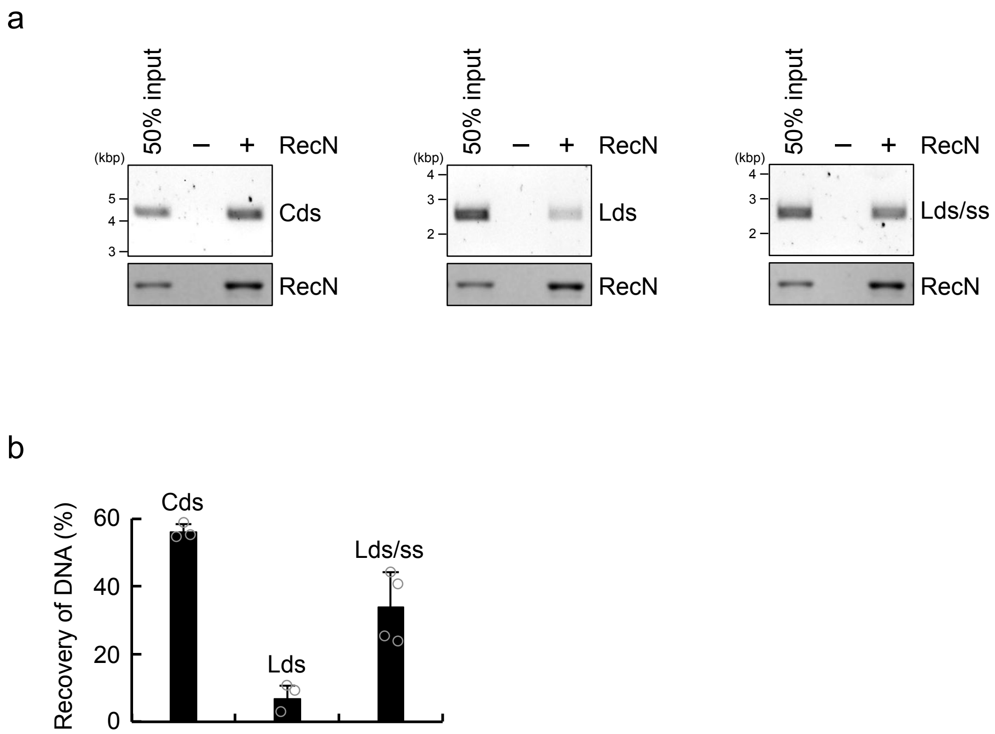

Supplementary Figure 5 The DNA-binding activity of RecN to linear dsDNA or linear ds/ssDNA.
a, The assays were performed using the same method as described for Fig. 4a,b. RecN was incubated at 37°C for 10 min in the presence of the following pUC19-derivative DNA substrates: circular dsDNA (Cds), linear blunt-ended dsDNA (Lds), and linear ds/ssDNA (Lds/ss). The RecN-DNA complexes on Co^{2+}-conjugated beads were collected at time zero of additional incubation without RecA and eluted with SDS-sample buffer. The eluted proteins were analyzed by SDS-PAGE and CBB staining, and the eluted DNA was analyzed by agarose gel electrophoresis and SYBR Gold staining. **b,** Quantification of the intensities of the DNA bands in the agarose gel images shown in **a**. Data represent the mean ± standard deviation of three independent experiments.

Supplementary Figure 6

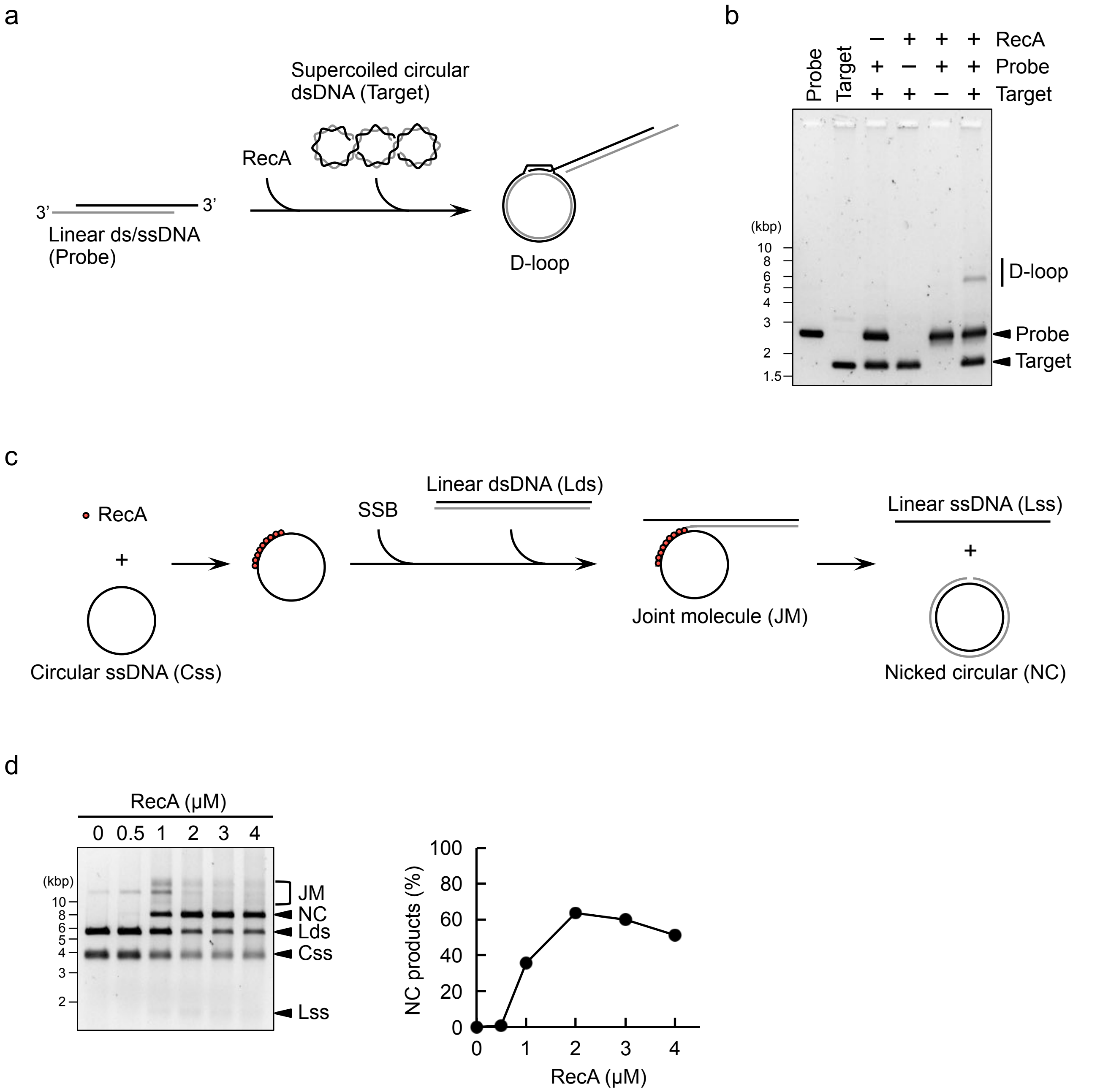

Supplementary Figure 6 RecA-mediated D-loop formation and strand exchange reaction.

a, Schematic illustration of the D-loop assay. RecA filaments formed on the linear ds/ssDNA (probe) containing 3' ssDNA overhangs promote homologous pairing of the ssDNA regions with a complementary region of the supercoiled circular dsDNA (target), resulting in formation of a D-loop structure. **b,** The dependency of D-loop formation in the presence of probe DNA (pUC19-derivative linear ds/ssDNA) and homologous target DNA (pUC19 supercoiled circular dsDNA) on RecA (2 μM) in incubation at 37˚C for 5 min. **c,** Schematic illustration of the RecA-mediated strand exchange reaction. A three-strand exchange reaction between homologous circular ssDNA (Css) and linear dsDNA (Lds) molecules produces joint molecules (JM), nicked circular dsDNA (NC), and displaced linear ssDNA (Lss). The reaction depends on the formation of a RecA filament on Css to promote homologous pairing, and on trapping of the resultant Lss by ssDNA-binding protein (SSB) to inhibit the reverse reaction. **d,** The formation of NC DNA (phiX174 derivatives), the final product, at varying concentrations of RecA in incubation at 37˚C for 90 min.

Supplementary Figure 7

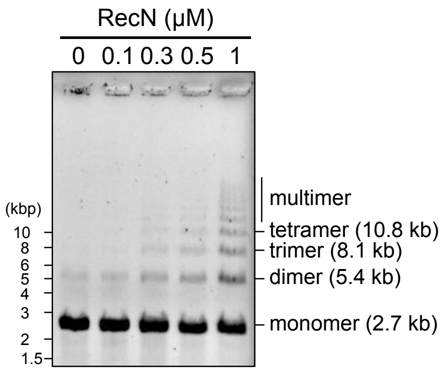

Supplementary Figure 7 The end-joining activity of RecN.
DNA ligation assays. The indicated concentrations (0–1 μM) of RecN were incubated with a pUC19-derivative blunt-ended linear dsDNA substrate (2.7 kb), prior to the addition of T4 DNA ligase. The DNA was purified and analyzed by agarose gel electrophoresis and ethidium bromide staining. The lengths of the linear multimeric products relative to the DNA size markers are shown as dimers (5.4 kb), trimers (8.1 kb), tetramers (10.8 kb), etc.

Supplementary Figure 8A

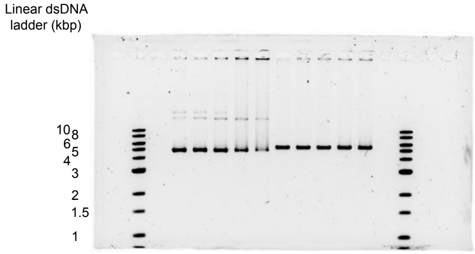

Full-length agarose gel from figure 1a

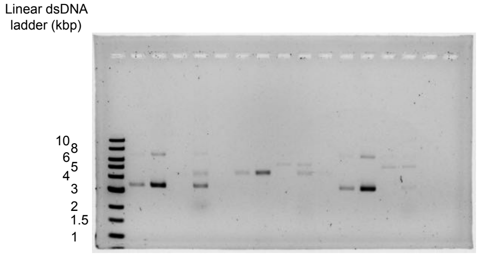

Full-length agarose gel from figures 2b and 2e

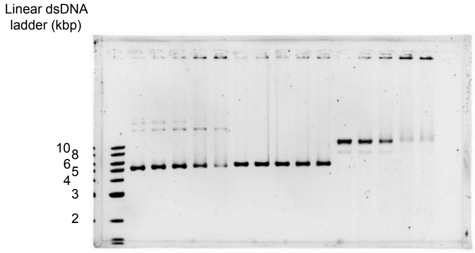

Full-length agarose gel from figure 1a

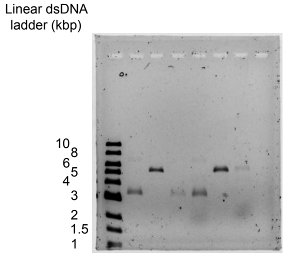

Full-length agarose gel from figure 2d

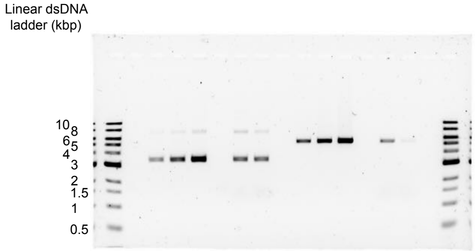

Full-length agarose gel from figure 1c

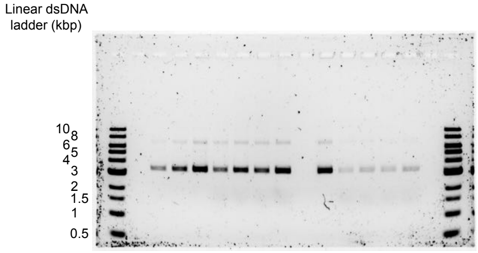

Full-length agarose gel from figure 3a

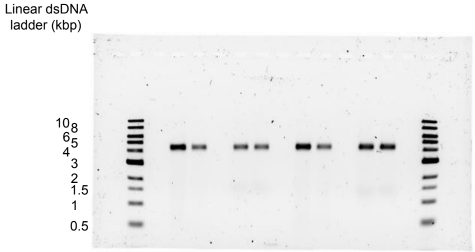

Full-length agarose gel from figure 1c

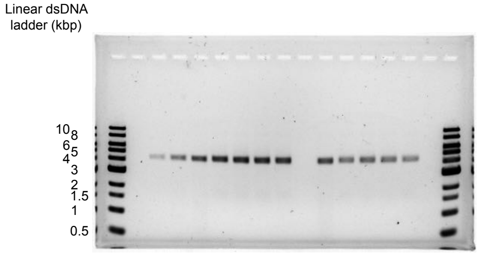

Full-length agarose gel from figure 3c

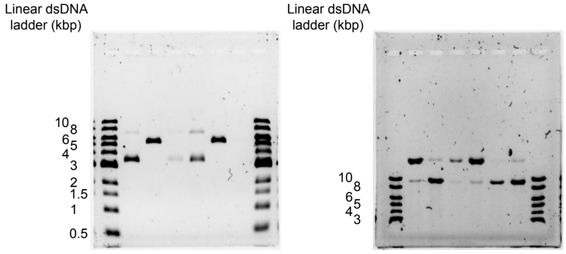

Full-length agarose gels from figures 1f and 1g

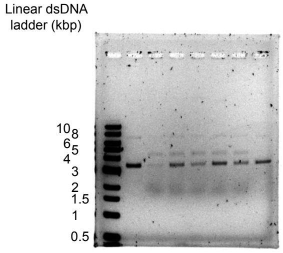

Full-length agarose gel from figure 3f

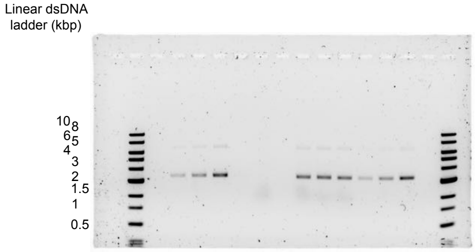

Full-length agarose gel from figure 2a

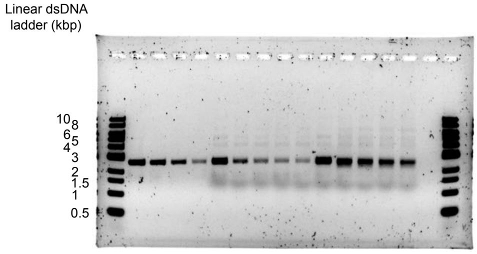

Full-length agarose gel from figure 4b

Supplementary Figure 8A Full-length gels presented in the figures of the paper.
The related figures of the paper are indicated.

Supplementary Figure 8B

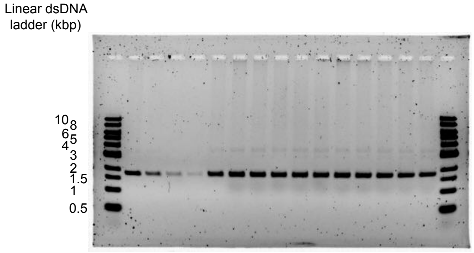

Full-length agarose gel from figure 4c

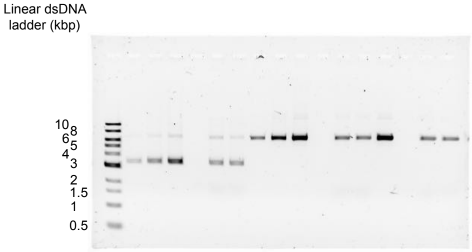

Full-length agarose gel from supplementary figure 1c

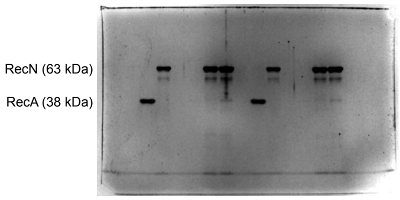

Full-length polyacrylmide gel from figure 4e

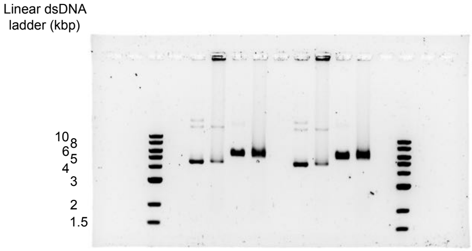

Full-length agarose gel from supplementary figure 1e

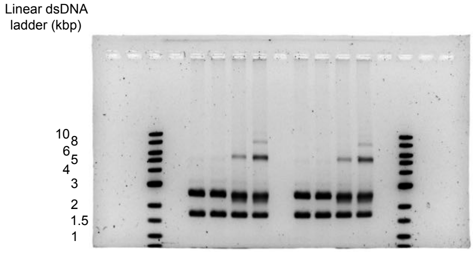

Full-length agarose gel from figure 5a

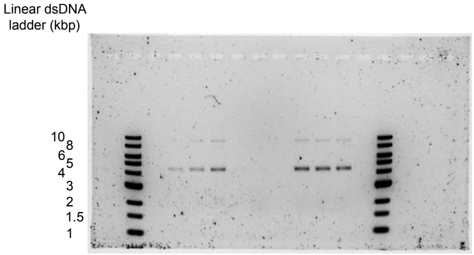

Full-length agarose gel from supplementary figure 3

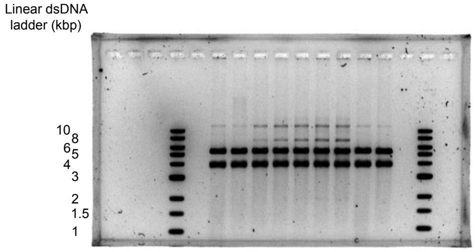

Full-length agarose gel from figure 5c

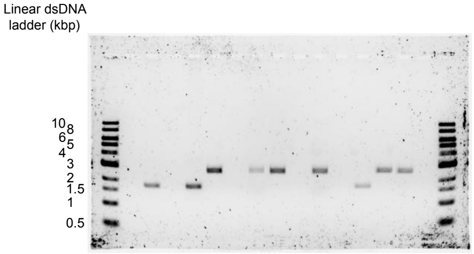

Full-length agarose gel from supplementary figure 5a

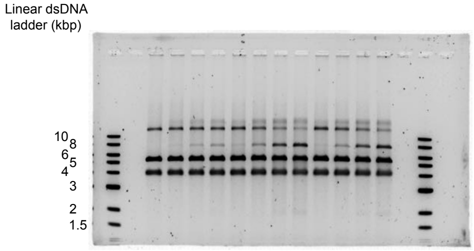

Full-length agarose gel from figure 5e

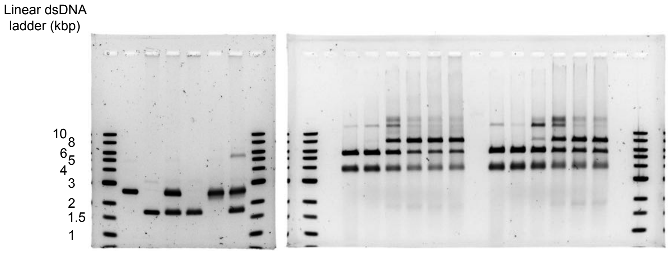

Full-length agarose gels from supplementary figures 6b and 6d

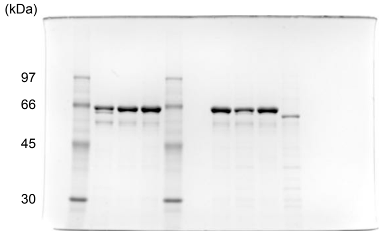

Full-length polyacrylamide gel from supplementary figure 1a

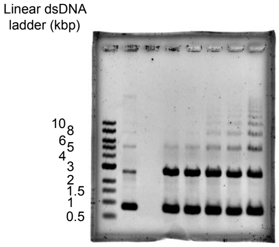

Full-length agarose gel from supplementary figure 7

Supplementary Figure 8B Full-length gels presented in the figures of the paper.
The related figures of the paper are indicated.
